# Regulatory Compliance of Health Claims on Omega-3 Fatty Acid Food Supplements

**DOI:** 10.3390/foods14010067

**Published:** 2024-12-29

**Authors:** Jelena Banović Fuentes, Ivana Beara, Ljilja Torović

**Affiliations:** 1 Department of Pharmacy, Faculty of Medicine, University of Novi Sad, 21000 Novi Sad, Serbia; 990d15@mf.uns.ac.rs; 2 Department of Chemistry, Biochemistry and Environmental Protection, Faculty of Science, University of Novi Sad, 21000 Novi Sad, Serbia; ivana.beara@dh.uns.ac.rs; 3 Center for Medical and Pharmaceutical Investigations and Quality Control, Faculty of Medicine, University of Novi Sad, 21000 Novi Sad, Serbia

**Keywords:** consumer information, food labelling, public health, supplementation

## Abstract

Unlike the mandatory information that must be provided on a food supplement label, health claims are voluntary. This study assesses the regulatory compliance of omega-3 fatty acid (ω-3-FA) supplement label claims. Of the 97 supplements, 76 (78.4%) carried verbal claims referring to active substance, of which 68 (89.5%) were claims specific to ω-3-FA. According to the European Union Health Claims Registry, 107 claims listed on 59 supplements were authorized, as opposed to nine unauthorized claims on nine supplements. The degree to which claims aligned with regulatory standards, expressed in terms of scores scaled from 0 to 1, was the highest for supplements intended for pregnant women (1), while, in the case of adults, the mean score was 0.76 ± 0.35, and, in case of children, was 0.85 ± 0.27. Statistical analysis revealed a minor tendency for higher health claim scores to be associated with lower product prices. Furthermore, differences in compliance levels across groups of supplements formed according to the country of origin/sources of ω-3-FA/target populations were explored. The main differences were associated with products from Sweden and Italy/fish oil/supplements for pregnant women. A comparison of the daily intake of ω-3-FA provided by the supplement (based on labeled information) with the intake requirements for supplements with claims referring to ω-3-FA, supported 91 claims, five were unsubstantiated, and 11 were missing required data. Supplements also contained mineral- (19 approved) and vitamin-related claims (73, of which 9 were unauthorized). This study’s findings reveal the extent of misuse of labelled information in markets that require pre-market supplement registration. Importantly, the non-compliance of health claims can significantly undermine consumer trust.

## 1. Introduction

### 1.1. Dietary Intake and Sources of Omega-3 Fatty Acids

A prospective cohort study that followed a large number of participants over many years in order to elucidate relations between dietary fats and total and cause-specific mortality identified health benefits associated with replacing saturated fatty acids with unsaturated fats and avoiding partially hydrogenated plant oils, which are sources of *trans* fatty acids [1]. In recent years, there has been a noticeable trend of decreasing intake of total and saturated fatty acids, accompanied by an increase in omega-6 fatty acid (ω-6-FA) but also a decrease in omega-3 fatty acid (ω-3-FA) intake, resulting in an ω-6/ω-3 ratio of 20:1 or higher. This trend has contributed to the rising prevalence of coronary heart disease, hypertension, diabetes, obesity, autoimmune diseases, cancer, and rheumatoid arthritis [2,3,4]. The intake of eicosapentaenoic acid (EPA) and docosahexaenoic acid (DHA), as well as alpha-linolenic acid (ALA), is suboptimal for a significant portion of the European population. A systematic review of 53 different studies conducted across 17 European countries between 2000 and 2015 revealed that population’s intake of EPA and/or DHA met recommended levels in only 26% of cases, while ALA intake was adequate in 77% of the countries [5].

The primary dietary sources of EPA and DHA are fatty fish such as salmon, tuna, herring, and mackerel. ALA, found in plant oils, serves as a precursor to EPA and DHA; however, its conversion efficiency in the human body is low, so the intake of preformed EPA and DHA is recommended [6]. An effective and relatively rapid strategy to counteract unfavorable intake trends may involve the fortification of foods with ω-3-FA. Notably, such fortified products are increasingly available on the market [7]. Additionally, supplements provide another source of EPA and DHA, with varying concentrations [6].

The European Food Safety Authority (EFSA) recommends daily intakes of EPA and DHA for European adults of between 250 and 500 mg/day, based on consideration of the risk of cardiovascular disease [8]. EFSA has defined reference values for nutrient intake for a healthy population, with values for specific nutrients varying by sex and age. According to these recommendations, the intake of linoleic acid (LA) and ALA is expressed as a percentage of total energy (E) intake, with recommended values of 5 E% and 0.4 E%, respectively, for the general population. For infants and children up to 2 years old, the recommendation is 100 mg of DHA per day; for children over 2 years old and adults, the recommendation is 250 mg of DHA + EPA. Pregnant and breastfeeding women are advised to consume an additional 100–200 mg of DHA daily, surpassing the standard recommendations for adults [9].

The amount of EPA and DHA in the body can be estimated by measuring the levels of EPA + DHA in red blood cells. This is expressed as the omega-3 index, which represents the amount of ω-3-FA consumed through food and supplements and it has the potential to be used as a factor for assessing the risk of coronary heart disease [10].

### 1.2. Health Significance of Omega-3 Fatty Acids

The primary health benefits of ω-3-FA vary depending on the population group. For pregnant and breastfeeding women, their intake is crucial for the proper development of the foetus and breastfeeding infants, who receive essential nutrients exclusively or predominantly through breast milk during the first six months. Additionally, recent recommendations suggest supplementing infants from birth with ω-3-FA due to their proven effectiveness in brain and vision development. The use of EPA and DHA during the first trimester of pregnancy is associated with significant increases in foetal growth [11], while supplementation throughout pregnancy and breastfeeding may offer benefits such as reducing the risk of preterm birth and low birth weight, preeclampsia, postpartum depression, and improving the newborn’s immune and visual status [12]. In a study conducted in Colombia, breastfeeding mothers received supplements containing 250 mg of DHA and 100 mg of EPA for three months, which resulted in a sufficient amount of DHA being transferred to the milk to meet the needs of the infants [13]. DHA is important for neurodevelopment during the foetal period and the first year of life as the most important fatty acid in the brain [14,15]. Although a systematic review confirmed the beneficial effect of ω-3-FA supplementation in pregnant and breastfeeding women on the positive cognitive development of children, the heterogeneity of the studies limited the establishment of a recommended dose for health benefits, and the impact on other developmental outcomes remains undetermined [16].

The beneficial effects of ω-3-FA in the adult population primarily impact cardiovascular health by protecting the heart and blood vessels, as well as preventing the development of atherosclerotic conditions caused by elevated cholesterol and triglyceride levels [17,18]. EPA and DHA modulate various risk factors for cardiovascular diseases, including blood lipids, blood pressure, heart rate, platelet aggregation, inflammatory processes, and endothelial function [19].

Observational studies have shown that higher serum levels of ω-3-FA are associated with a lower incidence of adverse cardiovascular events [20]. Supplementation with ω-3-FA in doses of from 2 to 4 g per day reduces triglyceride levels by from 25 to 40% in most patients, thereby providing a cardioprotective effect [18]. In the REDUCE-IT study, high-dose EPA supplementation as an adjunct to statin therapy led to a 25% reduction in the incidence of cardiovascular events in patients with elevated triglyceride levels [17]. A Japanese study was the first to introduce the concept of using purified EPA in the treatment and prevention of cardiovascular diseases. After five years, an intake of 1.8 g/day of EPA resulted in a significant 19% reduction in cardiovascular outcomes compared to statins alone [21]. However, this study had several significant limitations. The VITAL study followed participants free of cardiovascular disease at the start of this study, of which 37% were also on statin therapy, over 5.3 years. Participants were divided in two groups, one taking 840 mg/day of EPA and DHA and the other receiving a placebo. This study found no significant benefit of ω-3-FA supplementation in reducing cardiovascular events or cancer incidence [22]. Similarly, the ASCEND study investigated the same dose (840 mg/day of EPA and DHA) but focused on patients with diabetes mellitus without pre-existing cardiovascular disease. It was concluded that there was no difference between the ω-3-FA group and the placebo group, with 74% of participants concurrently using statins [23]. The U.S. Food and Drug Administration (FDA) has approved four formulations of ω-3-FA (2–4 g/day) as adjunctive therapy for reducing triglyceride levels [18]. On the other hand, high doses of ω-3-FA can lead to adverse effects such as atrial fibrillation [24,25] and bleeding (EPA supplements) [24].

A review of 78 randomized controlled trials found that only 43.6% of the studies demonstrated positive effects of ω-3-FA intake on cognitive function compared to placebo. However, this analysis included individuals without cognitive impairments, and the assessment tests used were not uniform [26]. It has been shown that patients who received 1 g of fish oil daily, either alone or in combination with vitamin E or carotenoids, experienced improvements in memory and mood [27,28].

Despite the well-known and long-exploited health benefits of ω-3-FA, their potential has not yet been fully utilized. The pandemic that began at the end of 2019 led to new recommendations regarding the intake of ω-3-FA. The potentially beneficial effects of ω-3-FA on acute respiratory distress syndrome (ARDS), which occurs in about 10% of patients infected with the SARS-CoV-2 virus and often has a fatal outcome, have been demonstrated. These effects include reducing the duration of the inflammatory process due to the anti-inflammatory properties of ω-3-FA, decreasing viral entry into cells, and affecting viral replication processes [29]. It was also hypothesized that the beneficial effects could be due to the anticoagulant properties of ω-3-FA [30]. Supplementation with ω-3-FA has shown a 12–21% reduction in the risk of contracting SARS-CoV-2. Patients with a deficiency in ω-3-FA experienced more severe disease and higher mortality rates. However, it has also been shown that increased concentrations of these fatty acids in the lungs of patients are associated with a higher risk of fatal outcomes [31].

The most important factors influencing consumers’ decisions to use food supplements are potential health benefits and safety. Information about health benefits can be found on the supplement labels, accompanying user information, and advertising materials (radio, print, television, internet), often in the form of health claims. Barriers to supplement use generally include a lack of belief in the health benefits, insufficient knowledge about the active ingredient, and high cost [32].

### 1.3. Regulations on Food Supplements and Health Claims

The European Union (EU) regulations related to food supplements have been transposed through harmonization process in the laws of candidate countries for joining the EU, such as the Republic of Serbia and the Republic of Srpska. Food supplements fall under the category of food as defined by Regulation 178/2002 [33]. Directive 2002/46/EC [34] defines supplements as “foodstuffs intended to supplement the normal diet and which constitute concentrated sources of nutrients or other substances with a nutritional or physiological effect, taken alone or in combination” and prescribes some general labelling requirements as outlined in (Check) Table 1.

Specific requirements for the labelling, advertising, and presenting of food (supplements) are part of Regulation (EU) 1169/2011 [35], which sets out mandatory information and specifies that voluntary information must be based on scientific evidence, as well as Regulation (EU) 1924/2006 [36], which governs the use of nutritional and health claims. Health claims are defined as any statement or claim that declares, suggests, or implies a relationship between a category of food, a specific food, or one of its ingredients and human health. A list of approved and unapproved health claims is available in the EU Register of Nutrition and Health Claims on the official EU website, presenting the type of health claim, the nutrient/substance/food/food category, the approved health claim, the conditions for using the health claim, and any warnings related to the health claim. These claims are included in Regulation (EU) 432/2012, which established a list of health claims that can be made on food, excluding those related to disease risk reduction intended for adults [37]. Additionally, Regulation (EU) No 440/2011 established a list of health claims related to the development and health of children [38].

The significance of Regulation (EU) 1924/2006 lies in protecting consumers by ensuring the availability of safe food that is properly labelled, including the use of nutritional and health claims [36]. Health claims, along with the market availability and financial affordability of supplements, can significantly influence consumer purchasing decisions. Given the increasing use of supplements due to anticipated health benefits [32], it is crucial that decisions about taking and choosing supplements are made through informed decision-making. The question is, do consumers understand and should they believe what are they told through health claims communicated on supplement labels? Thus, conceptual foundations for the current study include the theories underlying the regulatory science and practice of labelling.

From a practical aspect, this study’s initial question is “What can be found out regarding the food supplement health labelling based on the products’ labels?” (omitting analytical services and related financial demands). A foundation for such an approach can be found in the pre-market registration process of food supplements, which includes an administrative check of the supplements’ label but does not include verification of the presence/content of the active substances (as opposed to the substances of toxicological concern, included in safety regulations). The background questions are “To what extent supplement manufacturers comply with, exceed, or circumvent regulatory requirements?” and “Whether the mandatory pre-market registration fully protects consumers from misleading information, i.e., whether their trust is justified?”. To provide structure and focus to the investigation, a set of testable research questions is specified:

- “To which degree supplements bearing health claims align with regulatory standards?”;
- “What are the main predictors of regulatory compliance or non-compliance in supplement labelling?”;
- “Apart from the wording, are there any other issues related to the supplements’ compliance regarding health claims?”;
- “What is the value of ω-3-FA supplements measured through their contribution to the recommended dietary intake of ω-3-FA?”.

This study is designed to yield insights relevant to existing knowledge in the field that are informative for policy-making and industry investment. Thus, study findings should indicate whether a review of the existing regulatory framework and labelling practice is needed to support better communication between producers and consumers through health claims and other information presented on supplements’ labels, as well as interaction between producers and regulatory authorities. This study was conducted in the Republic of Serbia and the Republic of Srpska, where food safety regulations are harmonised with the legal framework in place in the EU. However, unlike the EU, they require pre-market registration of food supplements [39,40].

## 2. Materials and Methods

### 2.1. Collection of Omega-3 Fatty Acid Supplements

This study examined 97 unique products labelled as ω-3-FA supplements, representing 63 producers from 23 countries, obtained by a systematic search of the markets of the Republic of Serbia and the Republic of Srpska. One individual package of each ω-3-FA supplement was randomly selected among those available to consumers in pharmacies and specialized stores, purchased, and stored in the laboratory. Data were collected by reviewing the product labels and patient instructions (Supplementary Material Table S1). All available data were transferred to an Excel spreadsheet for further evaluation, which included the following information: product name, manufacturer name, type of raw material, country of origin of the product/raw material, active substance (s), quantity of active substance (s) per dose, recommended daily intake, target population groups, pharmaceutical form and number of dosage units per package, health claims for all active substances, and mandatory statements/warnings/limitations. Data were additionally accompanied with the price of each supplement. The price per daily dose of a supplement, calculated based on the price of one package of a supplement and the number of daily doses contained in one package, was considered to be the best measure for comparison between supplements from the consumers’ point of view.

The collection was assembled to provide insight into the representation of different pharmaceutical forms of supplements, as well as to examine selected characteristics of supplements categorized by target population groups (infants/children/adolescents/adults/pregnant and breastfeeding women) and sources of ω-3-FA (fish oil, algal oil, and plant-based oils). Key characteristics of omega-3 fatty acid supplements, namely, pharmaceutical form, source of ω-3-FA (raw material), target population group, and country of origin of supplements/raw materials, as well as price per daily dose, are presented in Appendix A.

### 2.2. Assessment of Regulatory Compliance of Health Claims/Mandatory Statements/Warnings/Restrictions on the Labels of Omega-3 Fatty Acid Supplements

Regulatory compliance of health claims listed on ω-3-FA supplement labels as well as compliance of ω-3 FA supplements themselves were assessed based on the following criteria:

(1) Are presented health claims in compliance with food regulations (authorization status—a binary view of compliance, yes or no)? ^(a)^
  The possibility of identifying key predictors of compliance or noncompliance was explored by assessing the relationship between regulatory compliance of ω-3 FA supplements and their country of origin/source of ω-3 FA/target population/price per daily dose.

(2) To what degree does each individual health claim presented on the supplement label/ supplement-bearing health claim (s) align with regulatory standards? For such a graded approach, a scale with a span from 0 (full non-compliance) to 1 (full compliance) was generated (e.g., an unauthorized claim referring to the healing of a disease was given a coefficient of 0; a claim related to the supplement in its entirety and not to the active substance was given a coefficient lower than 1, depending on the degree of alignment with an authorized claim referring to the active ingredient, etc.). The overall health claim score of a supplement was calculated as the mean value of individual scores of health claim (s) listed on the supplement’s label.
  To add further analytical depth, the correlation of compliance score with price per daily dose and significant differences in compliance scores across groups of supplements formed according to the country of origin/source of ω-3-FA/target population were explored. Groups with more than one supplement and groups with variable health claim scores were considered valid for analysis.

(3) Regardless of the wording of health claims, is the intake of ω-3-FA provided by the supplement in accordance with the defined conditions for supplements carrying health claims for ω-3-FA? ^(b)^

In order to gain more detailed and informative insight into the appropriateness of health claims, the following were additionally evaluated:

(4) To what extent does the intake of ω-3-FA from supplements contribute to the intake defined in the consumer information accompanying the health claim for ω-3-FA? ^(b), (c), (d), (e)^
(5) To what extent does the intake of ω-3-FA from supplements contribute to the recommended intake for the target population groups? ^(b), (f), (g), (h)^

^(a)^ The Regulations on nutrition and health claims of the Republic of Serbia [41] and the Republic of Srpska [42] are aligned with the requirements of EU Regulation (EU) 1169/2011 [35] and Regulation (EC) 1924/2006 [36]. The Register of Health Claims [43], established by these regulations, was used to assess the compliance (authorization) of health claims. The cited regulations were also used to evaluate the compliance of supplement labels concerning mandatory statements, warnings, and restrictions.

^(b)^ The intake of ω-3-FA from supplements was determined based on the labeled content of ω-3-FA per dosage unit and the recommended intake pattern (number of dosage units per day). The assessment was performed for supplements whose labels provided sufficient data to calculate the intake of ω-3-FA for the target population group. The conditions for supplements bearing health claims for ω-3-FA were derived from the Register of Health Claims [43].

^(c)^ For claims related to the benefits of DHA intake in mothers (normal brain and eye development of the fetus and infants), as well as the impact on blood pressure (EPA and DHA) and triglycerides (EPA and DHA; DHA), the amount of the active substance specified in the user information (required to achieve the health benefit) is consistent with the intake defined in the conditions for supplements bearing health claims for ω-3-FA (compliance assessment point (3)).

^(d)^ For other claims related to EPA and/or DHA, the intake defined by the conditions for supplements bearing health claims for ω-3-FA is consistent with the recommended daily intake. Therefore, the assessment according to this criterion was included in the evaluation of the supplement’s contribution to the recommended daily intake of ω-3-FA (informed insight point (5)).

^(e)^ Regarding the claim that ALA contributes to the maintenance of normal blood cholesterol levels, the difference between the intake specified in the user information and the intake defined by the conditions for supplements bearing health claims for ω-3-FA was considered. User information accompanying health claims for ω-3-FA was obtained from the Register of Health Claims [43].

^(f)^ The contribution is expressed as a percentage relative to the EFSA recommendations [9] for EPA and/or DHA intake for the target population groups.

^(g)^ Since EFSA recommendations for children start at 7 months of age [9], and some supplements for infants indicate use from 1–3 months (four supplements) or from 3 to 6 months (five supplements), for such cases the intake recommendation of 100 mg DHA daily for children aged 7–12 months was applied.

^(h)^ The assessment of intake was provided both for all supplements in their entirety and for supplements grouped by the source of ω-3-FA: fish oil, algae oil, and plant-based oil supplements.

Additional Note: in the case of labels translated from the original languages (imported supplements), translation errors were observed for a small number of supplements, such as an incorrect indication of the total net mass of the product. Additionally, for one supplement, there were discrepancies in dosing recommendations (data were taken from the translation as approved by the regulatory authorities). In cases where relevant data were not found in the translation, they were taken from the original label.

### 2.3. Statistical Analysis

The relationship between ω-3 FA supplement compliance with food regulations (a dependent variable) and their country of origin, source of ω-3 FA, target population, and price (independent variables) was investigated by regression analysis. Correlations between health claim scores and price per daily dose of a supplement were determined using the Pearson correlation test. Correlation coefficients were considered highly significant at *p* < 0.01. The differences between health scores of groups of supplements, formed according to the country of origin/source of ω-3 FA/target population, were evaluated using a *t*-test followed by a comparison of the means by the Tukey HSD test (*p* ≤ 0.05). All statistical analyses were performed using the STATISTICA version 14.0.015. (TIBCO Software Inc., Palo Alto, CA, USA).

## 3. Results

### 3.1. Health Claims on Omega-3 Fatty Acid Food Supplements

An overview of ω-3-FA supplements and authorized health claims presented on their labels (either individually or in combination) is provided in Supplementary Table S2.

**Figure 1:**
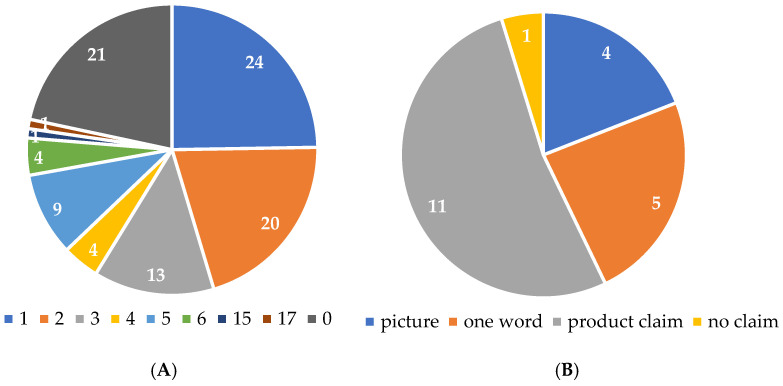
Number of omega-3 fatty acid supplements with: (**A**) marked number of verbal (in form of a sentence) health claims referring to the active substances; (**B**) health claims in form of pictures, one-word claims, claims referring to the supplement as a whole, or no health claims.

The majority of supplements (76 in total, 78.4%) featured verbal (in form of a sentence) health claims referring to the effects of the active substances (either one or more) (Figure 1A). Of the remaining 21 supplements, only one did not have any text, images, or graphics indicating health benefits; nine (9.3%) suggested health benefits with a single word or image (such as a heart, sun, or cross) which are not listed in the Register of health claims (Appendix B Table A1), and 11 (11.3%) included claims related to the supplement as a whole product (Appendix B Table A2), rather than to individual active substances (Figure 1B), which is not in compliance with regulations.

Additionally, two supplements presented both claims referring to the whole product and to active substance (s). Notably, within the group of supplements with verbal claims, there was a roughly equal representation of those with one (24:31.6%) or two claims (20:26.3%), together making up more than half of this group (57.9%). Two supplements with 15 and 17 claims, respectively, accounted for only 2.1% of all supplements but contributed with 14.5% of the total number of verbal claims.

Health claims referring to active substances other than ω-3-FA, recorded on 39 supplements (40.2%), primarily concerning minerals and vitamins and, to a much lesser extent, herbal ingredients, are presented and discussed in Appendix C.

### 3.2. Health Claims Referring to Omega-3 Fatty Acids—Regulatory Compliance

Health claims referring to ω-3-FA were listed on 68 out of 97 supplements (70.1%). A review of the supplements and health claims concerning ω-3-FA (either individually or in combination) is provided in Supplementary Table S2 (authorized) and Appendix B Table A3 (non-authorized).

**Figure 2:**
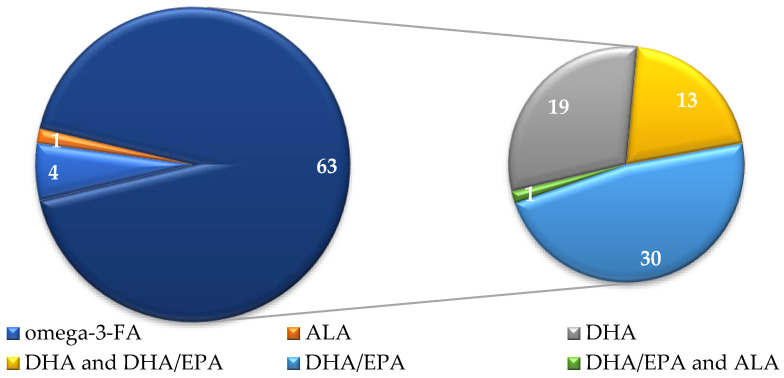
Distribution of supplements in relation to omega-3 fatty acid highlighted by the health claim (claims referring to EPA/DHA are represented in details in the small circle).

In terms of ω-3-FA types, the majority of supplements featured claims referring to DHA (63; 92.6% of supplements with ω-3-FA claims), whether DHA was the only FA highlighted (19) or the supplement included an integrated claim for DHA/EPA (44, including 13 combined with DHA claims) (Figure 2).

#### 3.2.1. Health Claims Referring to Omega-3 Fatty Acids—A Binary View of Regulatory Compliance

**Figure 3:**
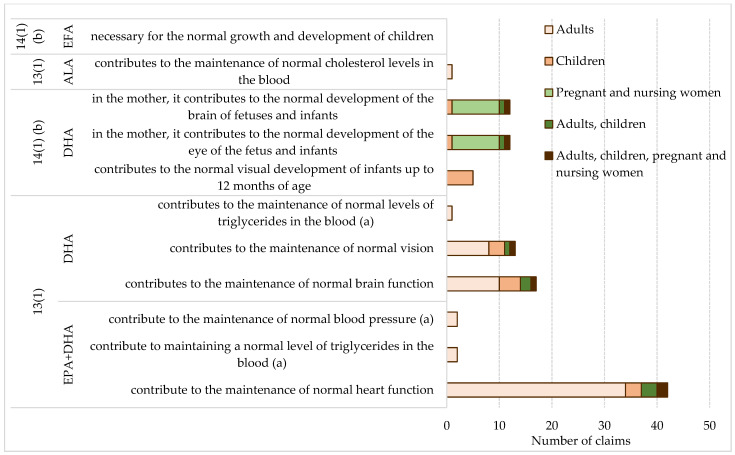
Authorized health claims referring to omega-3 fatty acids (EFA—essential FA, ALA—alpha-linolenic FA, EPA—eicosapentaenoic FA, DHA—docosahexaenoic FA) listed on supplements’ labels, categorized by the type of fatty acid and the regulation article to which the claim pertains (Article 13 (1)—general claims, Article 14 (1) (b)—claims related to children’s health) [43], displayed by the target population groups. ^(a)^ Usage restriction: The statement will not be used for food intended for children [43].

Of the 68 supplements with ω-3-FA health claims, 59 (86.8%) carried authorized claims [43], as shown in Figure 3, of which 31 (53.4%) had a single claim and 28 (47.4%) had two or more claims, totaling 107 individual authorized claims.

Among these 59 supplements, three (5.1%) had authorized ω-3-FA claims along with unauthorized claims for vitamins. A total of nine supplements carried unauthorized ω-3-FA claims (Appendix B Table A3), of which three were combined with authorized and an additional three with unauthorized claims referring to vitamins. In addition to two supplements originating from the Republic of Serbia and the Republic of Srpska, the non-compliant ones also included one imported from the USA, while the remaining were produced in the EU countries.

Considering the type of FA highlighted in the claim, one out of the 44 supplements with DHA/EPA-related claims carried an unauthorized claim (Appendix B Table A3), representing 2.3% of the supplements with DHA/EPA claims. Out of 32 supplements with claims specifically related to DHA as an individual active substance, three carried unauthorized claims that partially contained elements of authorized claims. Four supplements had claims that did not specify individual ω-3-FA but generally attributed health benefits to ω-3-FA. Out of the two health claims given for ALA, one was unauthorized (Appendix B Table A3).

An evaluation of health claims oriented toward the target population group is presented in Appendix D.

To analyze the relationship between ω-3-FA supplement compliance with food regulations considering health claims authorization status (compliant or non-compliant) and their country of origin, source of ω-3-FA, target population, and price per daily dose, regression analysis was undertaken (Table 2).

All R^2^ values were very low (<0.1), indicating that none of the individual predictors (country of origin, source of ω-3-FA, target population, or price) have significant explanatory power for compliance. This suggests that compliance is influenced by factors or interactions not captured in the current model. The low R^2^ values may also reflect the complexity of compliance determination, which could also involve other influences such as manufacturer reputation and labeling practices. In general, these findings highlight the need for further investigation and the incorporation of multifactorial variables to better understand the determinants of compliance. This approach could provide a more comprehensive understanding of the factors influencing compliance and improve predictive accuracy.

#### 3.2.2. Health Claims Referring to Omega-3 Fatty Acids—Regulatory Compliance Scores

**Figure 4:**
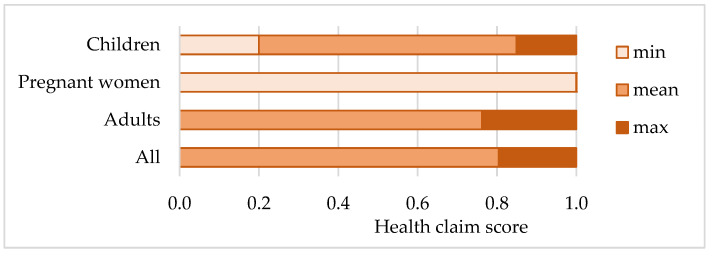
Health claim scores of omega-3 fatty acids supplements, displayed by the target population groups.

The health claim scores of supplements, based on the evaluation of the degree of alignment of each individual health claim listed on the supplement’s label with regulatory standards, are presented in Figure 4. For the collection of ω-3-FA supplements in its entirety, the health claim scores covered the full range from 0 to 1 (mean ± STD = 0.80 ± 0.32), mostly reflecting the largest group of supplements intended for adults (0.76 ± 0.35), in case of supplements for children the scores ranged from 0.2 to 1 (0.85 ± 0.27), while each supplement for pregnant women was rated with the highest score (1; full regulatory compliance).

**Figure 5:**
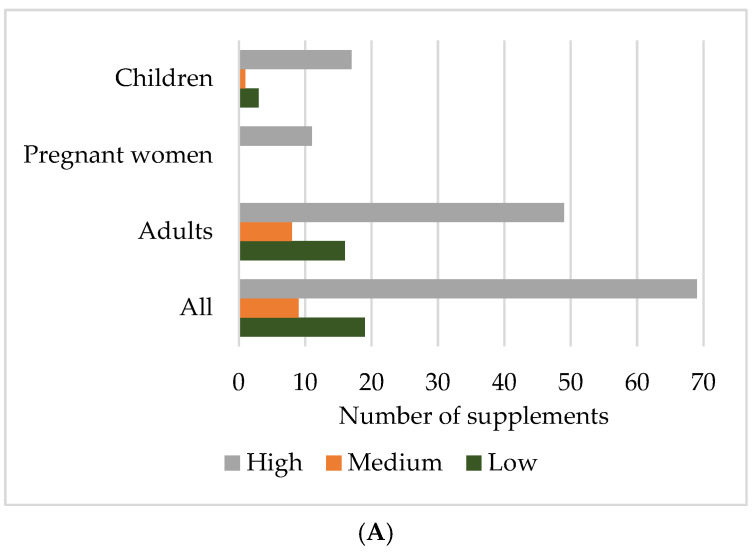

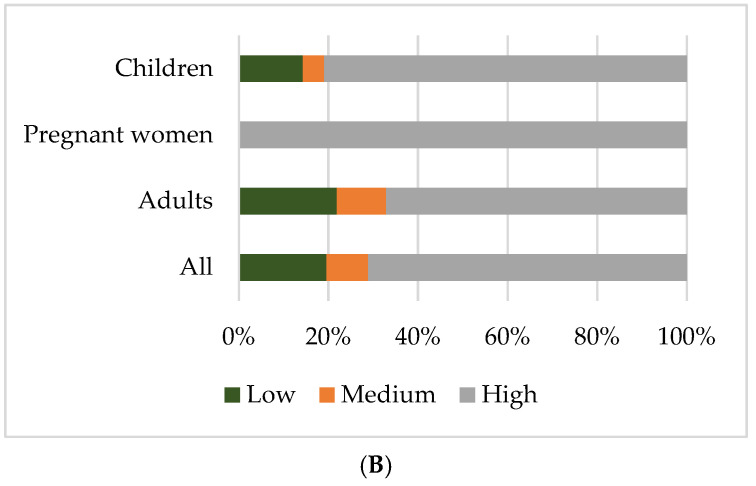
Classification of health claim scores of omega-3 fatty acids supplements, displayed by the target population groups (**A**) number and (**B**) percentage of supplements in a category.

Supplements’ health claim scores were further classified into low (0–0.330), medium (0.331–0.660), and high (0.661–1) groups. As can be seen in Figure 5, 100% of the supplements intended for pregnant women were attributed the highest score, followed by 81.0% of the supplements for children and 67.1% of the supplements for adults. Low scores were related mainly to the supplements for adults (21.9%) and, to a lesser extent, children (14.3%).

To add depth to the findings and allow for more robust conclusions, potential relationships between the health claim scores and selected characteristics of the supplements were explored.

Correlation with the price per daily dose, determined by calculation of Pearson’s linear correlation coefficients, is shown in Table 3, considering the target population. Across all groups, the relationship was weak (*R*^2^ values ranged from −0.284 to −0.298), indicating that the variables are not strongly correlated and that there is a minor tendency for higher health claim scores to be associated with lower price. In the group of supplements intended for pregnant women, the lack of variability in health claim scores (all claims/supplements fully compliant with regulations) precluded the calculation of a correlation coefficient.

The significance of differences in compliance levels (health claim scores) across supplements grouped by the country of origin/source of ω-3-FA/target population is shown in Supplementary Tables S3–S5, respectively. Regarding classification by country of origin, from Table S3, it can be observed that the main differences are associated with groups of products from Sweden and Italy, along with one instance of disparity between Norwegian and United Kingdom supplement groups. Specifically, health claim scores for Swedish products are statistically significantly different from those of almost all other European countries (except France, Poland, Slovenia, and UK), followed by the U.S. and Canada. Similarly, health claim scores for the Italian supplements group are statistically significantly different from those of supplement groups from nine other European countries and the U.S.

Regarding the source of ω-3-FA, it is notable that only the health claim scores for supplements derived from fish oil differ significantly from those derived from plant oil, while no significant differences were observed across all other supplement groups (Table S4).

With the highest compliance levels observed across all samples, the health claim scores for supplements intended for pregnant women differ significantly from those intended for general adult use (*p* = 0.0291). However, no significant differences were observed between the health claim scores of supplements for pregnant women and those for children, nor between products for adults and children (Table S5).

#### 3.2.3. Assessment of the Contribution of Intake of Omega-3 Fatty Acids from Supplements Relative to the Intake Requirements Defined by Regulations for Supplements Bearing Claims for Omega-3 Fatty Acids

**Figure 6:**
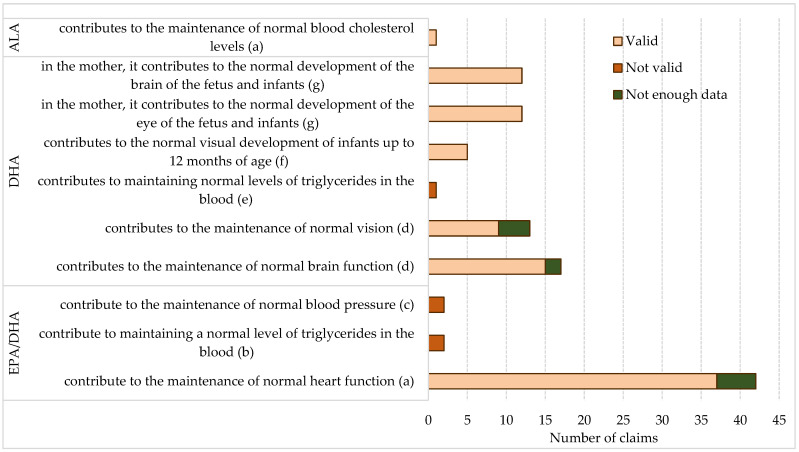
Validity assessment of health claims based on ω-3-FA intake from supplements in relation to the intake requirements for supplements bearing omega-3-FA health claims [43]. Notes: ^(a)^ A food that is at least a source of omega-3 fatty acids. A claim that a food is a source of omega-3 fatty acids, or any statement likely to have the same meaning for consumers, can only be made if the product contains at least 0.3 g of ALA per 100 g and per 100 kcal, or at least 40 mg of the combined EPA and DHA per 100 g and per 100 kcal. ^(b)^ A food that provides a daily intake of 2 g of EPA and DHA. ^(c)^ A food that provides a daily intake of 3 g of EPA and DHA. ^(d)^ Food that contains at least 40 mg of DHA per 100 g and per 100 kcal. ^(e)^ Food that provides a daily intake of 2 g of DHA and contains DHA in combination with EPA. ^(f)^ Food must contain at least 0.3% of its total fatty acids as DHA. ^(g)^ Food that provides a daily intake of at least 200 mg of DHA.

The validity assessment of health claims based on ω-3-FA intake from supplements relative to the intake requirements defined for foods with ω-3-FA health claims [43] is presented in Figure 6.

The validity assessment supported 91 out of 107 claims (85.0%) and deemed five (4.7%) as unsubstantiated, while, for 11 claims, there was no sufficient data. Considering the type of ω-3-FA highlighted by the health claim, the statement about the contribution of EPA/DHA to the normal maintenance of heart function, present in 42 supplements, is supported by the EPA/DHA content in 88.1% of them. Two supplements that make claims about the contribution of EPA/DHA to blood triglyceride levels and blood pressure provide only 10% and 15% of the required intake of these ω-3-FA. The claim about DHA’s impact on blood triglyceride levels is not substantiated, as the only supplement making this claim provides only 6% of the required DHA amount. Alongside these claims, it is necessary to include a warning regarding the safe intake level: the additional daily intake of combined EPA/DHA should not exceed 5 g, which is adhered to in both cases. The claim regarding DHA’s contribution to visual development in children up to 12 months old is substantiated for all five supplements on which it was made. These supplements are exclusively intended for children; three contained only DHA, while two had a combination of EPA/DHA, with DHA constituting 36.9% and 39.8%, respectively.

#### 3.2.4. Assessment of the Contribution of Omega-3 Fatty Acids Intake from Supplements Relative to the Intake Defined in the Consumer Information Accompanying Health Claims for Omega-3 Fatty Acid

Supplements carrying authorized health claims for ω-3-FA (59 out of 97) were assessed for their contribution to ω-3-FA intake relative to the intake defined in the consumer information accompanying the health claim for ω-3-FA. The supplement with the claim that ALA contributes to maintaining normal blood cholesterol levels provides only 16% of the intake defined in the consumer information that accompanies the ω-3-FA claims (this effect is achieved with a daily intake of 2 g of ALA through food and food supplements). This is in contrast to the full contribution to the intake defined for foods with ω-3-FA claims (foods that are at least a source of ALA) (Figure 6).

For health claims (specified in Section 2.2) where the intake of the active substance in the consumer information accompanying the ω-3-FA health claim matches, the following is true:

- The intake defined in the conditions for supplements carrying ω-3-FA claims, the assessment of contribution is provided in Section 3.2.3.
- The recommended daily intake for target population groups (claims related to EPA and DHA, excluding those specified in Section 2.2), the contribution assessment is provided in Section 3.2.5., evaluating all supplements, regardless of whether they carry ω-3-FA health claim (s) or not. However, since it can reasonably be assumed that the presence of a health claim increases the likelihood of a consumer choosing a supplement over others with the same active substance but without health claims, supplements that include ω-3-FA health claims are separated and evaluated as specific subgroups (Supplementary Comment S1).

#### 3.2.5. Assessment of the Contribution of Omega-3 Fatty Acids Intake from Supplements Relative to the Recommended Intake for Target Population Groups

**Figure 7:**
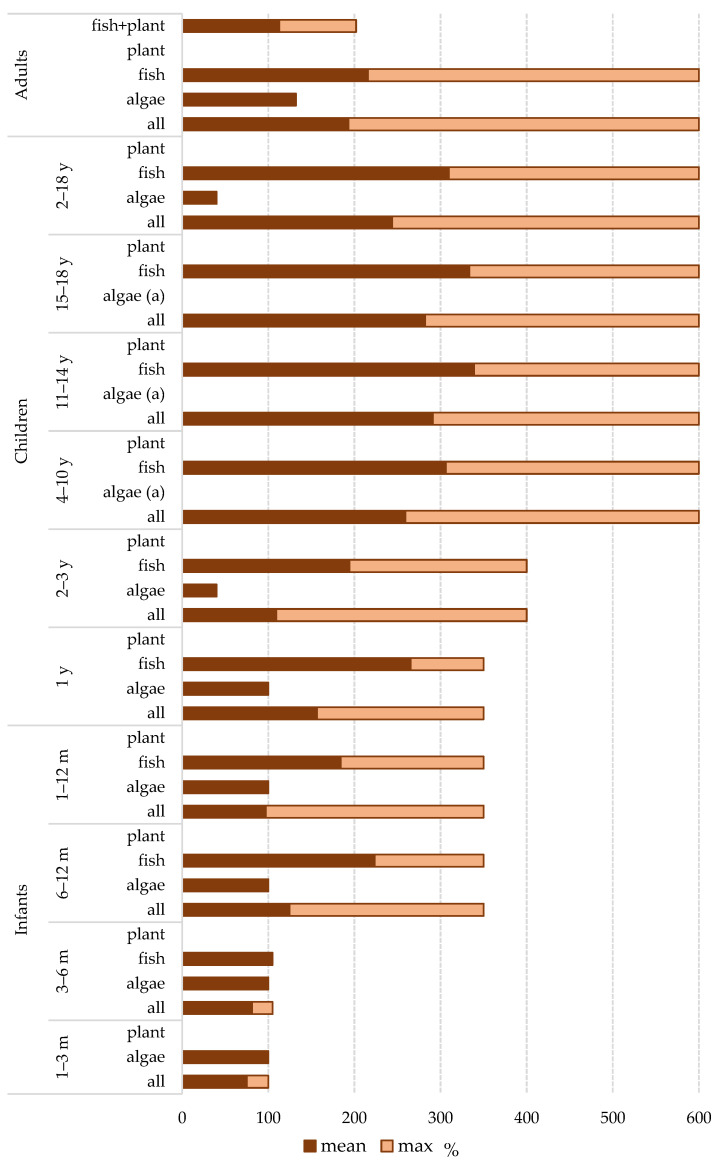
Percentage contribution of omega-3 fatty acid supplements relative to EFSA recommendations for EPA and/or DHA by age groups [9]. Note: ^(a)^—dosage of the supplement not labeled.

The assessment of the contribution of ω-3-FA intake from supplements relative to the recommended daily intake for target population groups [9] is shown in Figure 7 and Supplementary Table S6.

For children, daily intake recommendations vary by age. Of the eight supplements intended for children up to two years old, one (12.5%) was made from terrestrial plant oils and did not contain DHA. Algae-based supplements provided DHA amounts in line with the recommendations. For fish oil supplements, the contribution ranged from 97% to 350%. The average contribution of all supplements for children under two years was 116 ± 96%, with fish oil supplements providing an average contribution of 225 ± 111%, and algae oil supplements providing 100%. The average contribution of fish oil supplements in 3–6 months, 6–12 months, and 1-year age groups were 105 ± 0%, 224 ± 127%, and 265 ± 87%, respectively.

In the category of supplements intended for children from 2 to 18 of age, there were 21 supplements. Unlike two supplements made from terrestrial plant oils that do not contain EPA or DHA, supplements made from algae oil contributed a modest 40% of the recommended intake. In contrast, fish oil supplements showed a substantial contribution, ranging from 120% to 600%, with an average value of 310 ± 132%. When considering all supplements intended for children aged 2–18 years, regardless of the source of the raw material, the average contribution was 244 ± 170%.

Specifically, supplements intended for the age group of 2–3 years contributed on average with 109 ± 114%. In the overall group of supplements intended for the age ranges of 4–10 years, 11–14 years, and 15–18 years (regardless of the source), the results were very similar, with comparable average values of 259 ± 164%, 291 ± 163%, and 282 ± 166%, respectively. All fish oil supplements for age groups from 4 to 18 years provided an intake that fully met the recommendations, with average values of 306 ± 132%, 339 ± 120%, and 334 ± 124%, respectively.

Fish oil supplements intended for adults contributed to the recommended daily intake of EPA/DHA ranging from 23% to 600%, with an average of 216 ± 126%. Among the fish oil supplements, four failed to meet the minimum recommended values (23%, 60%, 72%, 93%), and one was on the borderline (99%). Supplements containing a combination of fish and plant oils had an average contribution of 113 ± 71% to the recommended daily intake, with individual values ranging from 27% to 202%. When considering all 73 supplements intended for adults, the average contribution to the recommended intake was 190 ± 126%. The majority of supplements (62, 84.9%) contained ω-3-FA in amounts sufficient to meet the recommended daily intake for adults.

Among the 11 supplements intended for pregnant women and breastfeeding mothers, the only algal oil supplement provided 240 mg or 480 mg of DHA, depending on the number of capsules consumed per day (recommended 1 or 2). All 10 fish oil supplements listed DHA content that provided between 200 and 300 mg of DHA, and seven also included information on EPA content (ranging from 40 to 300 mg). For five supplements (45.4%), the total EPA/DHA intake was below the recommended minimum of 250 mg (200–240 mg), while, for two additional ones, it did not reach the recommended levels of 250 mg EPA/DHA plus 100 mg DHA, or 250 mg EPA/DHA plus 200 mg DHA.

### 3.3. Mandatory Statements/Warnings/Restrictions

**Figure 8:**
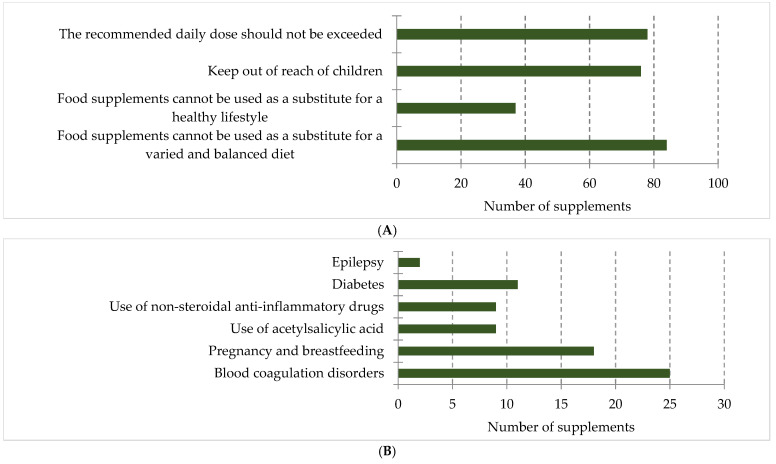
Information provided on supplement labels: (**A**) mandatory statements/warnings/restrictions; (**B**) reasons for consulting with a doctor or pharmacist.

The mandatory statements, warnings, restrictions, and notes provided on ω-3-FA supplements (Figure 8) were assessed in relation to the regulatory requirements.

The mandatory statement that supplements cannot be used as a substitute for a varied and balanced diet and a healthy lifestyle was included on 87 supplements (89.7%) (Figure 8A), of which 37 also included the statement that supplements cannot be used as a substitute for a healthy lifestyle. The mandatory warning “Keep out of reach of children” was absent from 21.6% and the advice not to exceed the recommended daily dose from 19.6% of the supplements. The warning that the supplement should not be used in children was present on 34 supplements. It is important to note that these warnings were specified for certain age groups (e.g., from 3 years, from 12 years, etc.), so eight supplements in this group were intended for specific age ranges only. Out of the 97 supplements, 44 (45.4%) carried warnings about the presence of allergens or sensitivity to certain ingredients, as well as the need to avoid use if an individual is allergic or sensitive to any of the components, or if an allergy or sensitivity occurs after consuming the supplement.

The warning requiring consultation with a doctor or pharmacist was present on 48 supplements (49.5%), with reasons for consultation varying from specific health conditions or diseases to medication use (Figure 8B). Four supplements included warnings for all potential users. The most common medical conditions prompting the need for consultation were blood clotting disorders as well as pregnancy and breastfeeding. Two supplements stated that there were no known side effects.

## 4. Discussion

The majority of 97 ω-3-FA supplements (78.4%) featured verbal claims about the health benefits of the active ingredients (one or more claims). Health-related single words and images (presented on 9.3% of the supplements), as well as claims referring to the product as a whole (11.3%), are not in compliance with regulations. The latter can mislead consumers into believing that health benefits are exclusive to that specific supplement, rather than achievable with any supplement containing the same active ingredients in equivalent amounts. This approach is a marketing attempt to highlight the superiority of one product over others. Summarizing the data, 89.7% of the supplements carried health claims, which is significantly higher compared to a similar study conducted in the Republic of Serbia more than a decade ago on vitamin–mineral supplements, where health claims were found on 46% of the products [44]. On the other hand, the result is comparable with a recent Serbian study conducted on herbal supplements, which revealed the presence of health claims on as much as 86.2% of the products [45], and a study conducted in the U.S. on fish oil supplements, where 73.9% of the products carried health claims [46]. Two supplements (2.1%), with 15 and 17 claims each, can be considered examples of over-use of health claims. However, the absence of markings that could be interpreted as beneficial effects might mislead consumers into perceiving the supplement as unnecessary or prompt discontinuation for economic reasons. This is particularly relevant considering that a portion of respondents in a recent survey conducted in the same region as the current study referred to the price of ω-3-FA supplements as a reason for not using them. Indeed, the price per daily dose of a ω-3-FA supplement was up to EUR 2.45 [47].

Given that the focus of this study was on ω-3-FA supplements, it was expected that the majority of health claims would be directed specifically at these active substances, namely, DHA and EPA in fish/crustacean oil or marine algae products, and ALA and GLA in terrestrial plant-based oil products. Indeed, the 68 supplements carried claims referring to ω-3-FA, of which 59 (86.8%) featured authorized claims (107 claims in total). Conversely, nine supplements displayed unauthorized claims for ω-3-FA, of which two triads featured combinations with authorized/unauthorized claims for vitamins. For comparison, a study in the U.S. found that 19.2% of ω-3-FA supplements carried qualified health claims approved by the FDA, while the remaining 80.8% carried structural–function claims, such as “promotes heart health” [46]. While official institutions in the U.S. maintain the position that consumers are not misled by health claims as long as they comply with FDA guidelines [48], some authors express doubts related to scientific evidence supporting the positive impact of fish oil supplements on heart health [49]. Additionally, among the 87 herbal supplements evaluated in a recent Serbian survey, those attributed with strictly prohibited properties of disease prevention/treatment/cure (9.3%), and those containing at least one botanical-related health claim not listed in the EFSA Register of questions, comprised even 50.6% [45]. Thus, in comparison with ω-3-FA, the market of herbal supplements exhibited a significantly less favorable situation, which can at least partly be explained by the extreme versatility of related claims and their on-hold status. Of the 44 ω-3-FA supplements with claims referring to EPA/DHA, 95.4% carried authorized claims, which is significantly higher than the 24% recorded in a study conducted in the U.S. on 46 ω-3-FA supplements [50]. Health claims related to cardiovascular benefits, specifically, heart and blood vessel health, were found on 59 supplements (86.8%) (with nine featuring unauthorized claims), making them the most common type of claim in the current study. A study conducted in the U.S. in 2022 on ω-3-FA supplements similarly reported that health claims most frequently target cardiovascular health (62.0%) [46], followed by brain and joint health. A recent study (2020) focused on the online marketing of cardioprotective supplements found that ω-3-FA supplements were the most common among these products (64.0%), with most of them featuring structural or functional claims (87.7%) and some also including risk reduction claims (40.4%) [51]. A similar study on the online marketing of cognitive enhancement supplements, conducted in 2023, revealed that ω-3-FAs were active ingredients in 12.0% of such supplements, though only one of them had ω-3-FA as the sole active ingredient [52].

From the perspective of target population groups, all supplements intended for use during pregnancy and breastfeeding carried only authorized claims. This finding is consistent with a Spanish study from 2023 that addressed and confirmed the adequacy of health claims related to selected micronutrients contained in supplements for pregnant women. The authors highlighted that although the composition of the Spanish supplements could justify the even higher number of claims, producers probably selected those that best aligned with marketing intentions [53]. In a binary view of compliance, the 17.6% proportion of non-compliant supplements intended for children due to unauthorized claims, either alone or combined with authorized ones, is considered exceptionally high, given that children represent one of the most sensitive population groups. When considering only claims related to ω-3-FA, this proportion is 20.0%. Among supplements intended for adults, 14.0% of the supplements with ω-3-FA claims, or 17.8% of all the supplements carrying claims, were non-compliant. In a non-binary approach, the degree to which claims aligned with regulatory standards, expressed in terms of scores scaled from 0 to 1, was the highest for supplements intended for pregnant women (1); in the case of adults, the mean score was significantly lower (0.76 ± 0.35), while supplements for children were somewhere in between (0.85 ± 0.27).

For a supplement to carry a specific claim, it must comply with the requirements for foods with health claims related to ω-3-FA, in terms of the quantity of active substances, i.e., ω-3-FA provided per intake. The assessment supported 85.0% of the health claims for ω-3-FA, including all claims presented on supplements for pregnant women, which is in agreement with the Spanish study on micronutrient supplements for pregnant women [53], while 4.7% of the claims were deemed unsubstantiated. When considering the type of ω-3-FA for which a health claim was made, out of the 42 supplements carrying a claim regarding EPA/DHA contributing to the normal maintenance of heart function, 88.1% justified the claim based on their EPA/DHA content. However, to achieve the necessary dose of EPA/DHA for contributing to the regulation of blood triglyceride levels (1.8–4 g DHA/EPA daily for a 13–19% reduction in triglyceride levels) [54,55], a greater number of daily doses would be required than indicated on the labels of the supplements (3) that carried such a claim. The doses recommended on the supplement labels are far below the study-validated doses for cardiovascular health benefits (2–4 g EPA/DHA per day), which could potentially have positive effects in terms of the primary and secondary prevention of cardiovascular outcomes [56]. Similar results were found in a 2021 study in the U.S. on fish oil supplements, based on the national supplement database, which determined that between 1 to 34 doses would be needed to achieve the required total dose of 2 g DHA/EPA [57]. Another study conducted in the U.S. in 2022 confirmed that a small proportion of supplements (9.4%) have the capacity to provide a daily dose of 2 g or more EPA/DHA [46].

Regarding the contribution to the recommended intake of ω-3-FA for target population groups, it is important to emphasize that supplements are intended to complement a varied and balanced diet, not serve as the sole source of nutrients, and are, therefore, not required to meet full daily nutritional needs. Nevertheless, it is noteworthy that supplements made from terrestrial plant oils do not contribute to the intake of EPA and DHA due to the absence of these FAs in terrestrial plants. For children, daily intake recommendations vary by age, and EFSA guidelines are provided for all nutrients starting from the seventh month of life when complementary foods are introduced alongside breast milk. However, supplements intended for infants include usage recommendations from 1–3 months (four supplements) and from 3–6 months (an additional five). Supplements for children are specifically categorized into those intended for children up to 2 years and those for older children. These supplements contributed between 0 and 350%, with an average of 116 ± 96%, and 0–600% with an average of 244 ± 170% of the recommended intake, respectively. The contribution from supplements for adults ranged from 0 to 600% with an average of 190 ± 125%. Among supplements intended for pregnant women and breastfeeding mothers, the total intake of EPA/DHA for five supplements was below the recommended minimum of 250 mg, and one more did not reach the level of 250 mg EPA/DHA + 100 mg DHA. A similar study conducted in the U.S. focused exclusively on fish oil supplements, found that EPA/DHA supplementation contributed 300–1100 mg daily, with a median of 600 mg/day (EPA doses ranged from 135 to 647 mg, and DHA from 140 to 500 mg) [46].

From the perspective of mandatory statements/warnings/limitations, it is important to emphasize that supplements should be clearly distinguished from medications on their labels to avoid misleading consumers. The statement that supplements cannot be used as a substitute for a healthy lifestyle suggests the need for physical activity and the abandonment of harmful habits, such as alcohol consumption, drug use, and smoking, in addition to a varied and balanced diet. This contributes to overall health and reduces the risk of disease. This statement was found in 86.6% of ω-3-FA supplements, which is higher than the 45.6% observed in a study conducted in Serbia in 2011 on vitamin and mineral supplements [44]. The warning that products should be kept out of reach of children was not included in 21.7% of supplements, despite this being a mandatory requirement. A similar study [52] revealed that 62.7% of cognitive enhancement supplements available for online sale lacked this warning. Supplements intended for children are often designed to be appealing in both form and taste to this age group; however, to prevent uncontrolled consumption, they should be kept in places inaccessible to children. The use of sweeteners to mask the taste is particularly pronounced in fish oil supplements, aiming to reduce children’s aversion to their consumption. The statement advising not to exceed the recommended daily dose was missing from 19.6% of supplements, which is significantly less than the 68.0% observed in the aforementioned study on cognitive enhancement supplements [52].

The statement recommending consultation with a doctor or pharmacist was found on 49.4% of supplements, which is also lower than the 69.3% observed in a study on cognitive function supplements [52]. Although they are not medications, supplements can have significant adverse health effects. This is suggested by studies indicating a link between ω-3-FA supplementation and an increased risk of atrial fibrillation and other cardiovascular events [58]. In 2.1% of cases, there was a direct statement that the supplement has no known adverse effects. However, such statements could mislead users into believing that the supplement is entirely safe. This conclusion aligns with a study on cognitive function supplements, where 5.3% of supplements explicitly claimed to have no negative effects [52].

### 4.1. Overall Considerations and Concluding Remarks

For consumers, two key factors in deciding whether to use a supplement are health benefits and safety [32]. A large study in the U.S. involving over 11,000 participants found that ω-3-FA supplements were among the top three types of supplements used, along with multivitamins and calcium supplements. Users of ω-3-FA supplements were often healthy individuals aiming to improve or maintain their health [59]. Additionally, a study conducted in the U.S. from 2015 to 2017 with cardiovascular patients found that the most common reasons for using ω-3-FA supplements were general health (34%), cardiovascular health (28%), coronary artery disease (12%), and lipid status disorders (8%) [60]. A survey conducted in 2023 among the general population of the Republic of Serbia and the Republic of Srpska highlighted the same reasons for the usage of ω-3-FA supplements [47].

The legal framework in the U.S. allows supplement manufacturers considerable freedom in marketing their products without having to provide evidence of safety and efficacy [46]. As a result, consumers may be at risk of replacing conventional therapies with supplements [61]. Most consumers lack sufficient information about clinical evidence supporting the effectiveness of supplements, and a limited understanding of the scientific literature can lead to skepticism toward clinical evidence. A survey conducted in the U.S. confirmed this trend, revealing that, for the majority of respondents, the decision to use supplements was influenced by recommendations from family, friends, or personal choice (86%), while recommendations from healthcare providers had a significantly lower impact (14%) [60].

A survey conducted in the EU entitled “Adapting Our Food for the Future—New Trends and Challenges” highlighted an increase in consumer interest in food information [62]. A special Eurobarometer survey conducted in the Republic of Serbia showed that consumers rely more on information exchange with family members, friends, colleagues, and social networks compared to the EU population, although television was the top source in both surveys. Additionally, Serbian people showed a significantly lower level of trust in all information sources, including healthcare professionals, scientists, and consumer organizations [63]. These findings were corroborated by a recent Republic of Serbia/Republic of Srpska survey, which focused on the use of ω-3-FA supplements [47].

The European Parliament’s resolution on the application of Regulation 1924/2006 concerning nutrition and health claims on food emphasizes that, even when health claims are scientifically based, their relevance must be considered [64]. Analysis of studies conducted in European countries across specific population groups (adults, children, pregnant/breastfeeding women) revealed that only 26% of the countries met the recommended intake levels of EPA/DHA from food [5]. Despite the heterogeneity in methodologies regarding the intake of EPA/DHA, whether through diet alone or with additional supplementation, such findings underscore the relevance of health claims for ω-3FA. This further highlights the need to ensure that the health-benefit information provided on the labels or any other form of presentation of supplements is accurate, scientifically based, and meaningful to consumers.

Studies examining compliance with supplement labeling regulations across different countries generally highlight rather common issues, as identified in the current study. The findings of this study reveal the extent to which all relevant parties adhere to regulatory requirements under given circumstances, i.e., labeling regulations, including mandatory pre-market registration of supplements. It is evident that there is room for improvement in the scope of the work of both supplement producers and the health authorities responsible for the registration and control of the supplements, both in terms of composition and labeling, including accuracy of health claims, all aimed at preventing misuse of information and maintaining consumers’ trust. However, evaluation of the possible predictors of claim non-compliance did not provide actionable insights; interestingly, even a high price of a supplement does not guarantee the accuracy of the presented claim (s). Yet, supplements for pregnant women seem to be more seriously treated regarding health claim labeling and/or control. It has to be stressed that non-compliance could substantially erode consumer confidence in health labels, supplements’ producers, and regulatory authorities, or potentially mislead consumers about product efficacy. Nevertheless, the question of how consumers understand and interpret statements on supplement labels and how these statements influence their decisions still remains without a comprehensive answer [64]. An analysis of consumer perceptions and understandings of health claims conducted in New Zealand through consumers’ participation in focus groups enabled the linking of five key themes with the roles that claims play as a component of decision-making: (1) aware of claims but did not use—the most influential drivers for food choice were price and habits; (2) mistrust and skepticism—perception by most of the health claims as marketing; (3) confusion and misinterpretation—consumers overwhelmed by so much different information on the package; a need for more contextual information; (4) using claims to guide food choice—a positive attitude among consumers with health issues; (5) not all claims are equal—the wording of a claim influences the interpretation of its credibility [65]. Such findings indicate the need for the creation of a trustworthy food label environment and education of consumers, otherwise, the utility of health claims will remain questionable. That can significantly impact the economic gain of producers who use health claims for product promotion but also can result in a lack of health benefits for restrained consumers.

### 4.2. Study Limitations and Future Directions

This study is designed to engage a broader academic audience through a theoretical evaluation of health-related labeling on food supplements, using only publicly available data provided on the product labels. While the analysis of the actual ω-3-FA content was conducted, comparing the labeled values with the determined content to verify ω-3-FA levels or detect potential fraud was beyond the scope of this study. An additional limitation is that this study focuses on a limited subset of one type of supplement (ω-3-FA) within a broad spectrum, relying solely on the information provided on the supplement itself. Consumers, however, may also access information through print media, television, internet marketing, sales, and social networks.

On the positive side, the gathered collection of 97 ω-3-FA supplements demonstrates significant diversity on multiple fronts, including a variety of pharmaceutical forms (capsules, liquids, candies, and gummies) and targeted consumer groups, covering all ages and specific groups such as pregnant and lactating women. It also encompassed typical sources of ω-3-FA from which supplements are derived (fish, algae, plants) and was highly diverse in terms of producers (63) and the countries of origin of the supplements (23), which supports the representativeness of this study findings.

To gain a comprehensive understanding of other influencing factors, such as manufacturer reputation and labeling practices, on health claims compliance, further investigation is required. For that purpose, a much broader spectrum of supplements from one manufacturer has to be considered, which implies the inclusion of supplements other than ω-3-FA, as manufacturers typically produce only one or a few supplements based on the same active ingredient. Furthermore, the outcomes of this study can inform future studies on consumer perceptions of health claims or the impact of different types of claims or claims compliance/non-compliance on consumer purchasing behavior and regulatory credibility. The confirmation of health claims compliance could be of substantial interest in strengthening consumers’ confidence and encouraging the use of claims as a useful tool for supporting better-informed purchasing choices. This study also provides practical implications for policymakers, regulators, and industry stakeholders, such as recommendations for stricter enforcement or improved labeling standards.

## Figures and Tables

**Table 1:** Regulatory requirements for food supplements labelling—mandatory items [34].

✓ Statement “nutritional supplement” or “food supplement” (along with the name of the supplement);
✓ Statement that food supplements cannot be used as a substitute for a varied diet;
✓ Names of the categories of nutrients or substances that the food supplement contains or a description of the nature of the nutrients or substances;
✓ Amount of food supplement recommended for daily intake;
✓ Warning for not exceeding the recommended daily dose; ✓ Warning for keeping out of the reach of children;
✓ Usage instructions;
✓ Usage restrictions and special warnings for potentially risky groups such as children, pregnant women, nursing women and individuals with chronic illnesses;
✓ Food supplements must not be presented as having properties for preventing, treating or curing diseases, nor can such properties be mentioned in the declaration, labelling and/or advertising of supplements.

**Table 2:** Regression analysis—predictors of compliance/non-compliance of omega-3 fatty acid supplements in relation to authorization status of presented health claims.

	The Country of Origin	The Source of Omega-3 Fatty Acids	The Target Populations	The Price of a Daily Dose
R^2^	0.008	0.009	0.052	0.046

**Table 3:** Pearson’s linear correlation coefficients between health claim scores and prices of omega-3 fatty acid supplements per daily dose, considering the target population.

Target Population	*R*^2^ (Health Claim Score vs. Price Per Daily Dose)
All (adults, children, pregnant women)	−0.284
Adults	−0.298
Children	−0.287
Pregnant women	/

**Figure A1:**
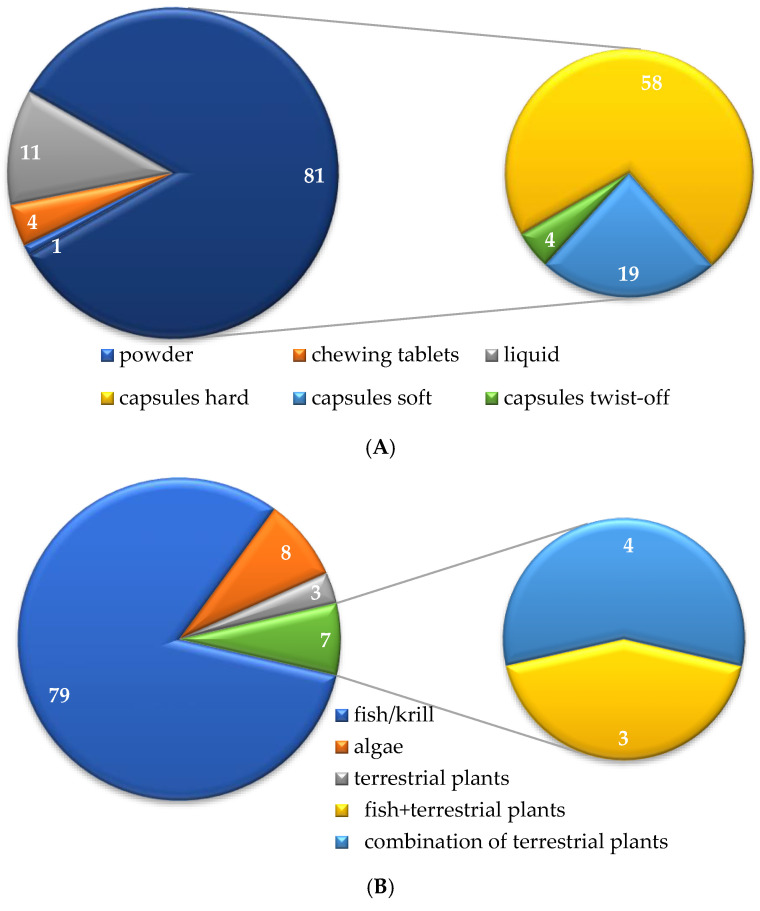

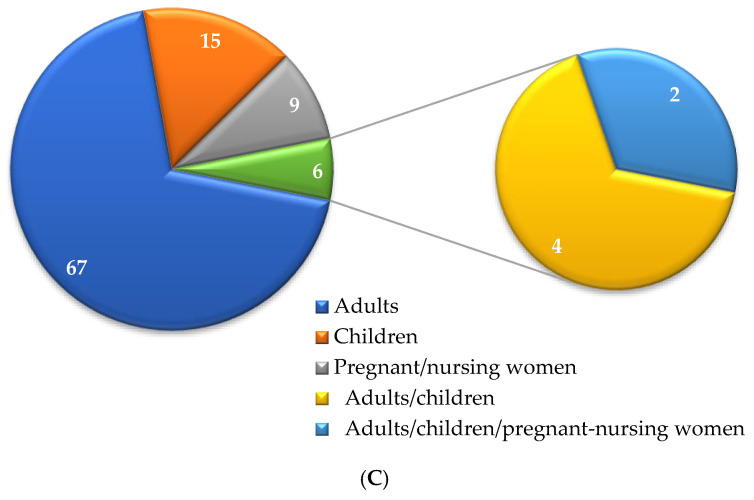
Distribution of omega-3 fatty acid supplements based on (**A**) pharmaceutical form (different types of capsules are represented in details in the small circle), (**B**) source of omega-3 fatty acids (combined sources are represented in details in the small circle), and (**C**) target population (combined target groups are represented in details in the small circle).

**Figure A2:**
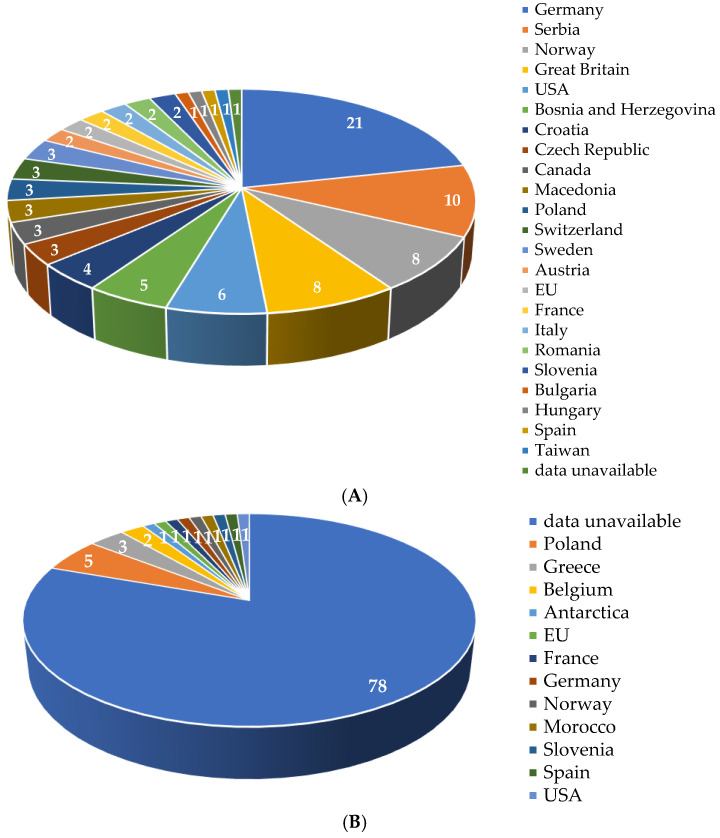
Distribution of omega-3 fatty acid supplements by country of production: (**A**) finished product, (**B**) raw material.

**Figure A3:**
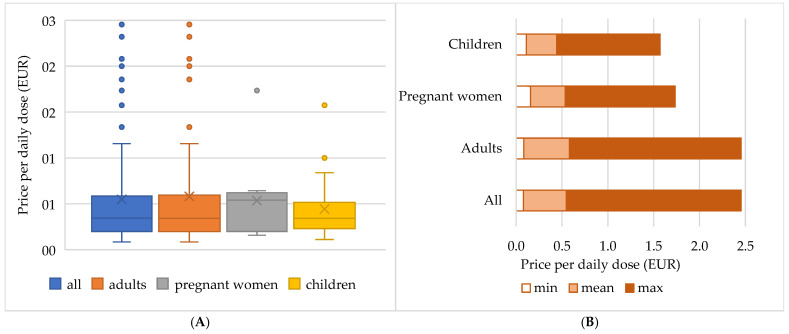
Distribution of omega-3 fatty acid supplements according to their prices per daily dose, displayed by the population groups: (**A**) Box–Whisker plot (whiskers extend from min to max, □ interquartile range, − median, × mean, ° outliers) and (**B**) range and mean.

**Figure A4:**
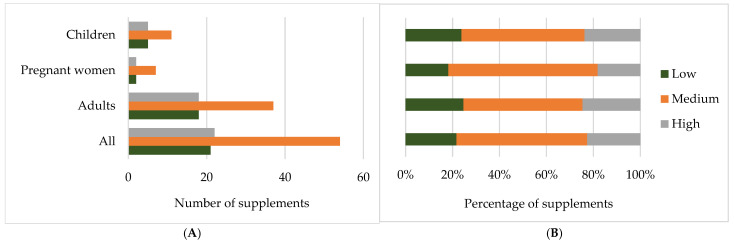
Classification of omega-3 fatty acid supplements according to their price per daily dose, displayed by the population groups: (**A**) number and (**B**) percentage of the supplements in a price category.

**Figure A5:**
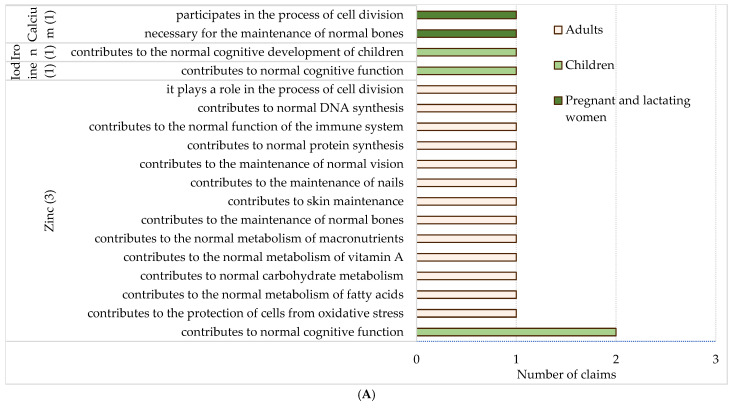

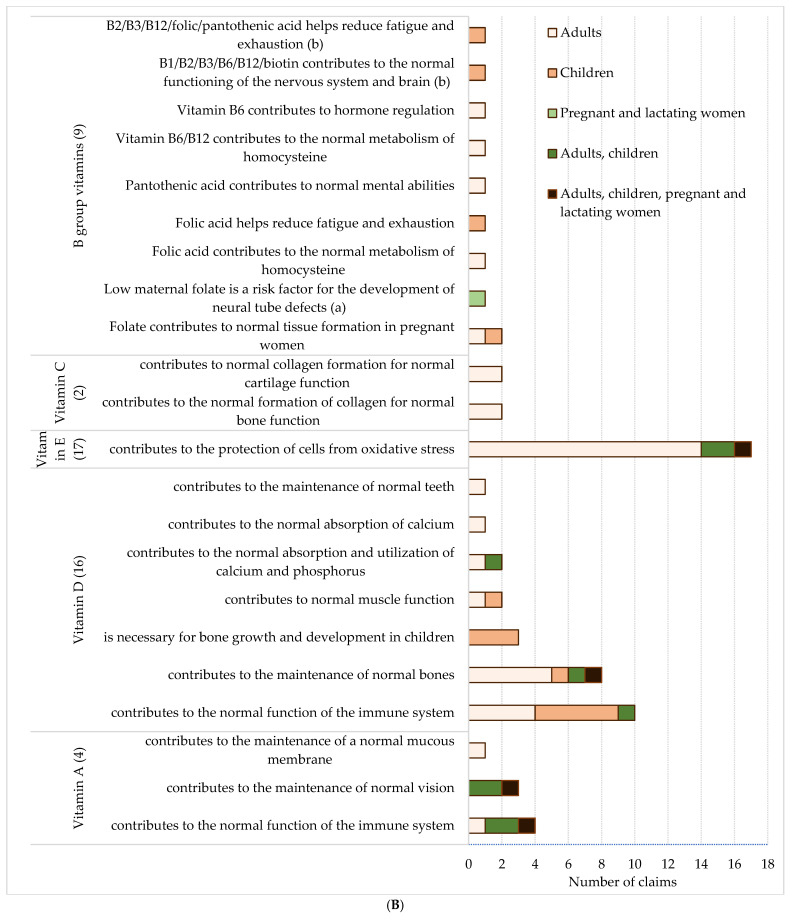
Authorized health claims listed on ω-3-FA supplements related to (**A**) minerals and (**B**) vitamins as active substances (distribution by the target population groups) [43]. ^(a)^ -In women of reproductive age, a beneficial effect is achieved with an additional daily intake of at least 400 µg. ^(b)^ The statement appears once for each of the listed vitamins individually. The number next to the name of the active substance indicates how many supplements feature the claim.

**Figure A6:**
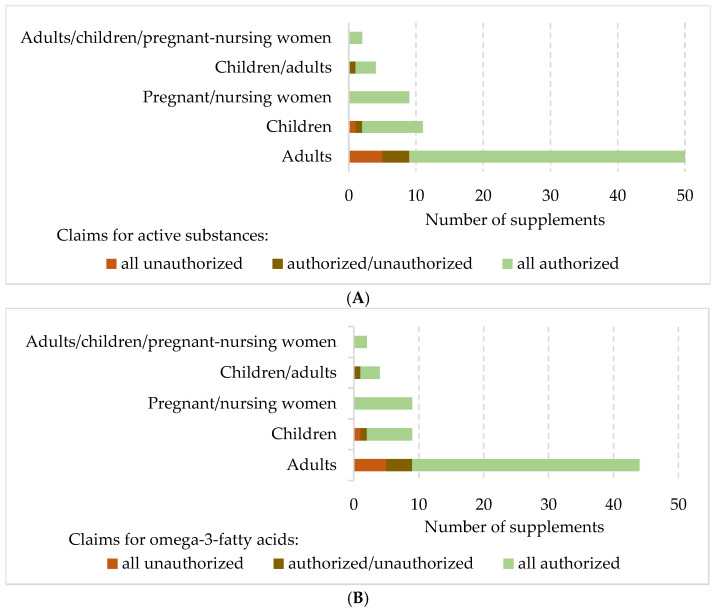
Distribution of omega-3 fatty acid supplements based on the authorization status of the claims they carry, shown by target population groups: (**A**) all health claims on the supplement, (**B**) health claims related to omega-3 fatty acids.

**Figure A7:**
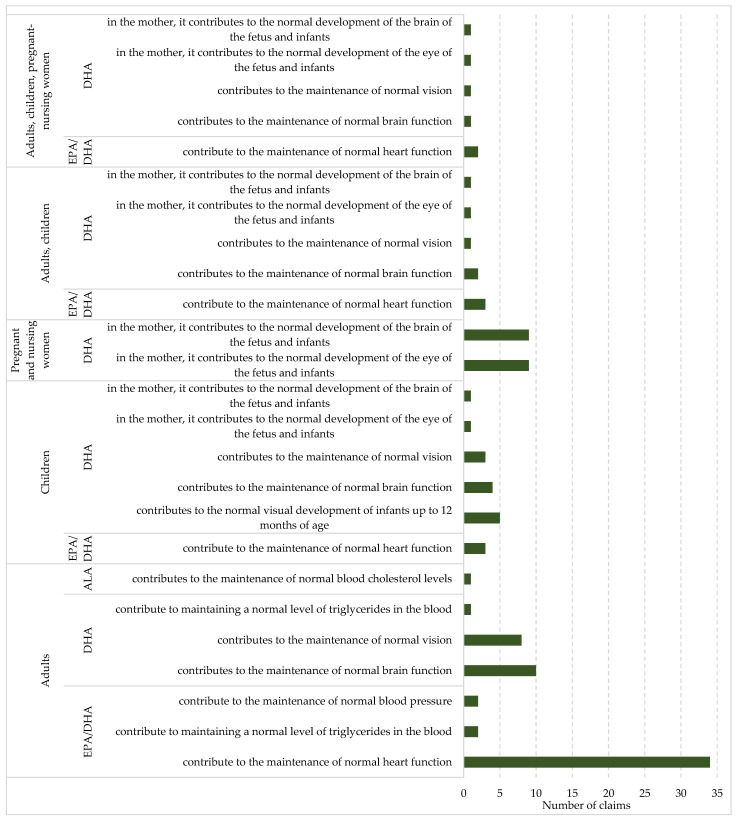
Distribution of authorized health claims referring to omega-3 fatty acids, in relation to the target population groups.

## Data Availability

The original contributions presented in this study are included in the article/Supplementary Materials. Further inquiries can be directed to the corresponding author.

## Supplementary Materials

The following supporting information can be downloaded at https://www.mdpi.com/article/10.3390/foods14010067/s1: Table S1: Data taken from omega-3 fatty acid food supplements labels; Table S2: Health claims referring to omega-3 fatty acids; Table S3: *t*-test *p*-values for statistical differences * in compliance levels (health claim scores) across the supplements grouped by the country of origin; Table S4: *t*-test *p*-values for statistical differences * in compliance levels (health claim scores) across the supplements grouped by the source of omega-3 fatty acids; Table S5: *t*-test *p*-values for statistical differences * in compliance levels (health claim scores) across the supplements grouped by the targeted populations; Table S6: Assessment of the omega-3 fatty acid intake expressed as percentage contribution in relation to recommended intake of EPA and DHA; Commentary S1: Assessment of the contribution of omega-3 fatty acids intake from supplements relative to the intake defined in the consumer information accompanying health claims.

## Appendix A

Key characteristics of omega-3 fatty acid food supplements

The gathered collection of ω-3-FA supplements demonstrates significant diversity on multiple fronts (Figure A1).

Pharmaceutical form of omega-3 fatty acid supplements

The majority of ω-3-FA supplements were in capsule form (83.5%), with hard gelatin capsules being three times more common than soft ones. Products specifically intended for infants were in the form of “Twist-off” capsules. Capsules have the advantage of easily masking the taste and odour, which might be off-putting to consumers. The physicochemical characteristics of ω-3-FA make it challenging to formulate supplements in solid dosage forms. However, modern technologies allow for the production of increasingly innovative forms with very high levels of active substances. Liquid supplements were substantially less common (11.3%), and only a few were available in chewable formulations, with those for children being in the form of caramels and candies, designed to be appealing to children and labelled as “nutraceuticals” (Figure A1A). The only supplement in powder form was a blend of hemp and flaxseed flour with spirulina, and its name indicated not only its rich ω-3-FA content but also a high protein content.

Sources of omega-3 fatty acids (raw supplement material)

All 97 supplements contained ω-3-FA as active ingredients. Among them, 42 contained ω-3-FA as the sole active ingredient, while the remaining 55 supplements included additional ingredients, the most commonly vitamins E (39), D (20), and B (14), as well as zinc (7).

The majority of supplements were made from a single source of ω-3-FA (90, 92.8%), while the remaining ones (7, 7.2%) contained a combination of multiple sources. Among these sources, pure fish/shrimp oils were predominant (81.4% in total, with 4.1% being shrimp oil), which is similar to the results of a study conducted in 2020, where 71.9% of ω-3-FA supplements available for online purchase were derived from fish oil [51]. Marine algae oils (8.2%) and terrestrial plant oils (3.1%) were much less represented. The combinations included mixtures of fish oils with plant oils or oils from different plant sources (Figure A1B).

Target population groups

Of the total 97 supplements, 21 were intended for paediatric use, with six of these also targeting adults, and two of those additionally intended for pregnant or breastfeeding women, each with different dosage recommendations. The distribution within the paediatric population included six supplements for infants (up to 1 year), 11 for young children (1–3 years), 16 for children (4–10 years), 16 for younger (11–14 years), and 15 for older adolescents (15–18 years). A total of 73 supplements were intended for the general adult population, and 11 for pregnant or breastfeeding women (Figure A1C).

Country of origin of omega-3 fatty acid supplements/raw materials

Only 15 supplements (15.5%) were produced in Republic of Serbia (10) and Republic of Srpska (5), while the majority came from other countries (84.5%), with one supplement lacking information on its origin. A total of 23 countries were listed as countries of production, with Germany (21), Serbia, Norway, and the United Kingdom being the most common (Figure A2A). Conversely, information on the origin of the raw materials was available for only 19 supplements, with only three countries appearing more than once (Poland, Greece, and Belgium) (Figure A2B).

Price per daily dose

Distribution of ω-3-FA supplements according to the price per daily dose is shown in Figure A3. For ω-3-FA supplements in their entirety, prices per daily dose ranged from EUR 0.08 to 2.45 (mean ± STD = 0.55 ± 0.56), mostly reflecting the largest group of supplements intended for adults (0.58 ± 0.62), in case of supplements for children the prices ranged from EUR 0.11 to 1.57 (0.44 ± 0.34), while prices of supplements for pregnant women ranged from EUR 0.16 to 1.74 per daily dose.

Supplements were further classified as low-, medium-, or high-price products. The criterion for classification was the following: low—from minimum to first quartile; medium—from first to third quartile; high—above third quartile level (according to the distribution of prices shown in Figure A3A, for each population group separately). The results of classification are shown in Figure A4. The highest share of supplements with high prices was recorded among supplements for adults and children (24.6 and 23.8%, respectively), while, in the case of pregnant women, 18.2% of supplements were considered high-price products.

## Appendix B

**Table A1:** Health claims listed on ω-3-FA supplements in the form of symbols and single words.

	Health Claims	N	Score
Image	The image of ***the heart*** emphasizes health	2	0.3
	The image of ***the sun*** emphasizes health	1	0.2
	The image of ***the cross*** emphasizes the health of the cardiovascular system	1	0.2
Word	The name ***WELLNESS*** emphasizes health	2	0.2
	The name ***IMUNO*** emphasizes the positive effect on immunity	1	0.2
	The name ***GOLD*** emphasizes the potential benefit	1	0.2
	The name ***POW(d)ER*** emphasizes the potential benefit	1	0.2

**Table A2:** Health claims listed on ω-3-FA supplements referring to the supplement as a whole product.

Health Claims	N	Score (s)
It contributes to maintaining the health of the cardiovascular system	1	0.5
It contributes to the support of the physiological health of infants and young children	1	0.3
It supports brain function, vision and heart health	1	0.3 + 0.3 + 0.3
For heart health	1	0.2
It supports the health of the cardiovascular system and brain function. It helps to reduce serum triglycerides. It helps conventional therapy to reduce rheumatoid arthritis pain in adults	1	0.3 + 0.3 + 0.2 + 0
Supporting joints and improving general health	1	0 + 0.1
It has a beneficial effect on the cardiovascular system. It affects the reduction of LDL cholesterol and triglycerides and the growth of HDL cholesterol, which reduces the possibility of blood clotting, thus protecting the heart and cardiovascular system	1	0.2 + 0.2 + 0 + 0.2
For maintaining normal functions of the cardiovascular, nervous and locomotor systems as well as preserving vitality	1	0.4 + 0.4 + 0.1 + 0.1
For dietary treatment in cancer patients	1	0
For dietary treatment of osteoarthritic changes in the joints	1	0
Fish oil has a beneficial effect on the skin and face, helps with acne-prone skin and psoriasis. Improves hair growth, regulates body weight. Protects heart, brain and joint constriction. It is considered a good remedy against nervous diseases, depression, improves vision. It is an excellent anti-aging agent and even anti-cancer because it regenerates the liver ^(a)^	1	0
Cardioprotective effect ^(b), (c)^	1	0.3
For the protection of the heart and blood vessels ^(c)^	1	0.4 + 0.2

Note: in addition to the above, the marked supplement also carried a statement for the active substance: ^(a)^ The most extreme example of non-compliant claims observed in one liquid fish oil supplement, which listed beneficial effects on various organs and systems (heart, brain, liver, joint), and directly mentioned diseases (psoriasis, nervous diseases, depression, cancer), conditions and processes such as aging, hair growth, and body weight regulation. ^(b)^ unauthorized statement for DHA, ^(c)^ authorized statement for vitamin E.

**Table A3:** Unauthorized health claims listed on ω-3-FA supplements referring to ω-3-FA.

Unauthorized Health Claims	N	Score (s)
*Claims referring to omega-3 fatty acids ^(a)^*		
Omega-3 fatty acids have a beneficial effect on the health of the cardiovascular system and on the level of triglycerides, total and LDL cholesterol	1	0.5 + 0.5
Omega-3 fatty acids have a beneficial effect on the functioning of the cardiovascular system, brain, immunity and improvement of dry skin	1	0.5 + 0.5 + 0.2 + 0.2
Omega-3 fatty acids support brain function, aiding normal cognitive function throughout all life stages	1	0.5 + 0.5
Omega-3 fatty acids have a beneficial effect on the elasticity of the sperm membrane, which is important for fertilization	1	0.25
*Claims referring to EPA/DHA*		
The use of EPA and DHA may reduce the risk of coronary heart disease. EPA and DHA support heart health	1	0.25 + 0.7
*Claims referring to DHA*		
Taking DHA contributes to the normal development of infants up to 12 months	1	0.75
DHA from algae supports brain development and function, aids normal cognitive function at all stages of life, promotes healthy visual function and heart health	1	0.5 + 0.5 + 0.5 + 0.5
DHA provides a positive influence on the normal functioning of the brain and vision	1	0.4
*Claims referring to ALA*		
Alpha-linolenic acid favours healthy development and supports cognitive functions	1	0.5

^(a)^ Three supplements were labelled that they contained EPA and DHA, while one did not specify a particular fatty acid but indicated that the source was fish oil.

## Appendix C

Health claims referring to active substances other than omega-3 fatty acids

Health claims referring to active substances other than ω-3-FA, recorded on 39 supplements, primarily concerned minerals and vitamins (vitamin E > D > B group) (Supplementary Table S2), and, to a lesser extent, herbal ingredients. Among these, eight supplements had claims exclusively referring to minerals and/or vitamins, while the remaining ones also included claims for ω-3-FA. Unauthorized claims for vitamins are listed in Table A4, while authorized claims for minerals and vitamins are shown in Figure A5A,B, respectively). All claims for minerals, including calcium, iron, zinc, and iodine, were authorized, with a notable occurrence of multiple claims related to zinc, with 14 different claims across only three supplements.

**Table A4:** Unauthorized claims listed on ω-3-FA supplements referring to vitamins as active substances.

Active Ingredient	Unauthorized Health Claim	N-Claims	Score (s)
Vitamin A (1)	Maintains cognitive functions	1	0.5
Vitamin D (1)	Contributes to the protection of cells from oxidative stress	1	0.25
Vitamin E (3)	A powerful antioxidant that protects the body from free radicals and has a beneficial effect on all conditions accompanied by oxidative stress	1	0.5
	Has a positive effect on cell metabolism and cell protection	1	0.25
	Maintains cognitive functions	1	0.5
B vitamins (3)	B group vitamins contribute to the normal function of the nervous system, normal mental work, etc.	1	0.25 + 0.25
	Vitamin B5 maintains cognitive functions	1	0.5
	Vitamin B6 maintains cognitive functions	1	0.5
	Vitamins of the B group contribute to normal psychological function, functioning of the nervous system and normal mental abilities	1	0.75

Note: (The number) next to the name of the active substance indicates how many supplements feature the claim.

The majority of vitamin-related claims pertained to vitamin E, with 17 supplements featuring the same authorized claim repeatedly (Figure A5B). Additionally, three other supplements had one claim each (Table A4), and none of these claims were fully compliant. Claims related to vitamin D were the next most common, found on 16 supplements. These claims were more varied compared to vitamin E, with seven different authorized claims appearing a total of 25 times (Figure A5B) and one unauthorized claim present on an additional supplement (Table A4). Claims regarding vitamin A were noted on four supplements, including three different authorized claims with a total of eight occurrences (Figure A5B) and one unauthorized claim on another supplement (Table A4). B vitamins featured 19 different authorized claims, with a total of 20 occurrences across nine supplements (Figure A5B), in contrast to three supplements with a total of four unauthorized claims (Table A4). Specifically, claims related to folates and calcium were associated with supplements intended for pregnant and breastfeeding women, while supplements for children carried claims about zinc, iron, and iodine.

Only two supplements (2.1%) featured a single claim related to plants: “Ginkgo leaf extract contributes to maintaining normal circulation and memory” or “Evening primrose contributes to skin health, hormonal health, and metabolic function regulation.” It is important to note that claims related to herbal supplements are categorized as “on-hold” claims (Regulation 432/2012) [37] whose authorization in the EU has not yet been completed.

## Appendix D

Health claims in relation to the target population groups

The distribution of supplements based on the authorization status of the health claims they carry, whether considering all health claims or only those related to ω-3-FA, is presented by the target population groups (Figure A6). Additionally, the distribution of health claims by the type of ω-3-FA, categorized by the target population groups, is presented in Figure A7.

All 11 supplements intended for use during pregnancy and breastfeeding carried authorized claims for active substances (Figure A6). Among the supplements exclusively targeting this population group, all nine stated the contribution of DHA intake to the normal development of the brain and eyes in foetuses and infants.

Among the 21 supplements intended for children, one did not carry any health claim, another had a non-verbal claim in the form of a sun image (Table A1), two included claims related to the supplement as a whole product (Table A2), and one each had authorized claims for iron and vitamin D. The remaining 15 supplements carried claims related to ω-3-FA. The statement “DHA intake contributes to the normal development of infants up to 12 months” is partially accurate but cannot be considered authorized because it does not specify the organ or system affected by the substance (i.e., it should specify that it contributes to the normal development of vision). Two such supplements also carried the complete statement “DHA contributes to the maintenance of normal brain and vision function”, so the incomplete statement listed after the complete one relies on the complete one, and thus these supplements are categorized as products with authorized claims. The statement “DHA enables a positive impact on the normal functioning of the brain and vision” does not fully meet the requirements for correctly defining DHA’s effect on the development and functioning of these organs. The term “positive impact” may be interpreted as implying superior effects, and it raises the question of whether DHA contributes to the effects of other components.

The proportion of non-compliant supplements due to unauthorized claims, either exclusively or in combination with authorized ones, was 17.6% of the total number of supplements with claims (3 out of 17), or 20.0% (3 out of 15) when considering only those with ω-3-FA claims (Figure A6A,B, respectively). One supplement with a non-compliant claim referring to ω-3-FA also had unauthorized claims for vitamin A (1) and B vitamins (2). Research by Hitl et al. [47] indicated that supplements for children are most frequently used based on medical recommendations, which should not endorse specific brands, and addressing issues with non-compliant supplements falls outside their responsibilities.

Among the 73 supplements intended for adults, eight (11.0%) did not carry verbal claims (Table A1), and 11 (15.1%) carried unauthorized claims related to the supplement as a whole product (Table A2). Of these, two had unauthorized claims such as “cardioprotective effects” and “for heart and blood vessel protection”, in addition to claims for individual active substances. Due to the simultaneous presence of authorized claims for vitamin E (in both supplements) and an unauthorized claim for DHA (in one supplement), these two supplements were included in the group with claims for individual active substances. Out of 56 such supplements, six (10.7%) did not have claims related to ω-3-FA but had authorized claims for vitamins A, D, E, C, and B vitamins. Among the 50 supplements with claims related to ω-3-FA, as many as 39 carried the authorized claim that “EPA and DHA contribute to the normal function of the heart”. A total of seven supplements carried unauthorized claims for ω-3-FA, suggesting beneficial effects on cognitive functions, immunity, conception, dermatological impact, and reduction of cardiovascular disease risk. Two of these also had authorized or unauthorized claims for vitamins, while three supplements had both authorized claims for ω-3-FA and unauthorized claims for vitamins. This results in a total of five supplements for adults with unauthorized claims for vitamins E (3), D (1), and B (2), amounting to a total of 10 supplements with non-compliant claims related to ω-3-FA and/or vitamins. The percentage of non-compliant supplements was 14.0% among those with claims for ω-3-FA and 17.8% among all supplements intended for adults.

It should be noted that claims defined in Article 13 (1) of the European regulation appear on supplements intended for both adults and children, as well as on those intended exclusively for children. However, the regulation defines claims related to children’s health with specific conditions given in Article 14 (1) (b). On the other hand, the restriction on claims regarding blood pressure (EPA/DHA) and triglycerides (EPA/DHA; DHA), stating that such claims should not be used on food intended for children [43], has been properly applied.

It is also notable that there is a health claim suggesting benefits from DHA intake for mothers on supplements intended for the general adult population. This can be explained by the use of supplements among women of childbearing age, potentially future mothers.
