# Supplementary material for: Regulatory Compliance of Health Claims on Omega-3 Fatty Acid Food Supplements
Source: Foods. 2024 Dec 29;14(1):67. doi: 10.3390/foods14010067 (PMC11719789; doi:10.3390/foods14010067)
Supplement: Supplementary file 1 [file foods-14-00067-s001.zip › foods-3387167-supplementary.pdf]

SUPPLEMENTARY MATERIAL

Regulatory Compliance of Health Claims on Omega-3 Fatty Acid Food Supplements

Jelena Banović Fuentes [1], Ivana Beara [2] and Ljilja Torović [1,3,*]

[1] Department of Pharmacy, Faculty of Medicine, University of Novi Sad, Novi Sad, Serbia; 990d15@mf.uns.ac.rs
[2] Department of Chemistry, Biochemistry and Environmental Protection, Faculty of Science, University of Novi Sad, Novi Sad, Serbia; ivana.beara@dh.uns.ac.rs
[3] Center for Medical and Pharmaceutical Investigations and Quality Control, Faculty of Medicine, University of Novi Sad, Novi Sad, Serbia; ljilja.torovic@mf.uns.ac.rs
* Correspondence: ljilja.torovic@mf.uns.ac.rs

**Supplementary material**			**Page**
**Tables**	Table S1	Data taken from omega-3 fatty acid food supplements labels	2
	Table S2	Health claims referring to omega-3 fatty acids	34
	Table S3	T-test *p*-values for statistical differences* in compliance levels (health claim scores) across the supplements grouped by the country of origin	39
	Table S4	T-test *p*-values for statistical differences* in compliance levels (health claim scores) across the supplements grouped by the source of omega-3 fatty acids	40
	Table S5	T-test *p*-values for statistical differences* in compliance levels (health claim scores) across the supplements grouped by the targeted populations	40
	Table S6	Assessment of the omega-3 fatty acid intake expressed as percentage contribution in relation to recommended intake of EPA and DHA	41
**Comment**	Comment S1	Assessment of the contribution of omega-3 fatty acids intake from supplements relative to the intake defined in the consumer information accompanying health claims for omega-3 fatty acids	42

Table S1. Data taken from omega-3 fatty acid food supplements labels

N	Product name	Manufac. name	Type of raw materi al	Country of origin – product (raw material)	OMEGA-3 (g/dose unit)	Other active ingredients	Recomm. daily intake for target population groups	Pharmac. form, N of dosage units per package, net mass	Health claims for all active substances	Statement(s)	Warning(s)
**1**	Krillol 500mg	Pharmavital	shrimp oil	Germany (Antartica)	EPA 0.06, DHA 0.0325	astaxanthin 100 mcg	1-3	capsules, 60, 42.9 g	EPA and DHA contribute to the normal function of the heart (a beneficial effect is achieved with a daily intake of 250 mg of EPA and DHA)	Supplement cannot be used as a substitute for a balanced and varied diet and a healthy lifestyle. Keep out of reach of children	The recommended daily allowance should not be exceeded
**2**	Baby guard® DHA	Evital	fish oil	Romania	DHA 0.15	Vitamin D 10 µg (400 IU)	2	twist-off capsules 30, 12.3 g	DHA contributes to maintenance of normal brain function. DHA intake contributes to the normal visual development of infants up to 12 months of age. Vitamin D contributes to maintaining the health of bones and teeth and to the normal functioning of the immune system	Intended for newborns	Caution when opening capsules. Make sure that the newborn does not swallow the capsule. Do not give in case of allergy to any ingredient of the product
**3**	OM3 Junior	SuperDiet	fish oil	France	EPA 0.228, DHA 0.072	Vitamin D 10 µg (400 IU)	children 6 to 9 years: 1-2, over 10 years: 2-3	capsules, 45, 33.5 g	*(There is no verbal or non-verbal claim)*	Take care of a varied and balanced diet and a healthy lifestyle. Keep out of the reach of small children	Recommended daily doses should not be exceeded. Not recommended for pregnant and lactating women
**4**	Omega-3 1000mg	Sunlife	salmon oil	Germany	EPA 0.18, DHA 0.12	Vitamin E 5 mg	1	capsules, 60, 83.1 g	EPA and DHA contribute to the normal function of the heart (a beneficial effect is achieved with a daily intake of 250 mg of EPA and DHA)	Supplements cannot be used as a substitute for a varied and balanced diet and a healthy lifestyle. Keep out of reach of children	Recommended daily doses should not be exceeded

**5**	Wellness pack man	Oriflame	fish oil	Sweden	EPA 0.075, DHA 0.05	/	2	capsules, 63	The name *WELLNESS* emphasizes health	Nutritional supplement should be a substitute for a balanced and varied diet. Keep out of reach of children. Do not use if the protective film is damaged or removed	Recommended daily doses should not be exceeded. Consult a doctor if you have a medical condition
**6**	Alkakaps omega 3	Alkaloid Skopje	fish oil	Macedonia (Poland)	EPA 0.135, DHA 0.09	Vitamin E 7.5 mg	2	capsules, 60, 42 g	EPA and DHA contribute to the normal function of the heart (a beneficial effect is achieved with a daily intake of 250 mg of EPA and DHA)	Supplement cannot be used as a substitute for a varied diet and a healthy lifestyle. Keep out of reach of children	The recommended daily dose must not be exceeded. Supplement is not intended for children and persons sensitive to its ingredients. Pregnant and lactating women should consult a doctor before use
**7**	Ecomer	Natumin Pharma AB	shark oil	Sweden	/	/	1-2 cps 2-3 times	capsules, 60, 24.5 g	The name *IMUNO* emphasizes the positive effect on immunity	A nutritional supplement is not a substitute for a balanced diet	The recommended daily dose must not be exceeded. Side effects are not known! The supplement is not intended for children, pregnant and lactating women
**8**	Omegactive	Esi	flax, evening primrose, olive, rice husks, blackthorn fruit oils	Italy	ALA 0.36, GLA 0.008	/	2 to 3	capsules, 120, 156 g	It contributes to maintaining the health of the cardiovascular system	Vegan! 100% herbal supplement in a special soft herbal capsule. Keep out of reach of children	/

**9**	Mivolis omega-3 1000	For Mivolis dm: Euro vital Pharma	fish oil and salmon oil	Romania	EPA 0.16, DHA 0.1	Vitamin E 12 mg	1 to 2	capsules, 60, 85.1 g	EPA and DHA contribute to the normal function of the heart (a beneficial effect is achieved with a daily intake of 250 mg of EPA and DHA). Vitamins of the B group contribute to normal psychological function, functioning of the nervous system and normal mental abilities. Vitamin E contributes to the protection of cells from oxidative stress	A nutritional supplement should not be a substitute for a balanced and varied diet. Keep out of reach of children	The prescribed dose must not be exceeded. The product should not be taken by people who are hypersensitive to any of the ingredients. Pregnant and lactating women should use the product with caution. Due to the possible interaction with drugs, other supplements or food, you should consult a doctor or pharmacist before use. People who have disorders with a tendency to bleeding, who are on warfarin therapy or suffer from haemophilia are recommended to consult before taking this product. It is not recommended to take the product for people who are preparing for surgical intervention
**10**	Dr Viton Omega-3	Alphacaps	fish oil	Germany	EPA 0.18, DHA 0.12	Vitamin E 12 mg, Vitamin D 10 µg	1	capsules, 60, 87g	EPA and DHA contribute to the normal function of the heart (a beneficial effect is achieved with a daily intake of 250 mg of EPA and DHA). Vitamin E contributes to the protection of cells from oxidative stress. Vitamin D contributes to the maintenance of normal bones. Vitamin D contributes to the normal absorption and utilization of calcium and phosphorus. Vitamin D contributes to normal muscle function	A nutritional supplement should not be a substitute for a balanced and varied diet. It is important to follow a balanced and varied diet and a healthy lifestyle. Keep out of reach of children	The recommended daily dose should not be exceeded. People who use drugs that affect blood clotting should consult a doctor before taking this supplement The supplement must not be used by people who are hypersensitive to any ingredient of the supplement

**11**	Norsk tran	Biopharma	cod liver oil	Norway	EPA 0.2, DHA 0.3	Vitamin E 6 mg, Vitamin D 20 µg, Vitamin A 250 µg	2.5 ml once a day for children from 3 and adults	liquid, 500 ml	EPA and DHA contribute to the normal function of the heart (a beneficial effect is achieved with a daily intake of 250 mg of EPA and DHA). Vitamin E contributes to the protection of cells from oxidative stress. Vitamin D contributes to the maintenance of normal bones. Vitamin A contributes to the maintenance of normal vision	A nutritional supplement should not be a substitute for a balanced and varied diet and a healthy lifestyle. Keep out of reach of children	The recommended daily dose must not be exceeded. Consult a doctor/nutritionist before use. People using anticoagulants should consult their doctor before using Norwegian omega-3 oil. Persons under the age of 18, pregnant and lactating women should use this product only upon recommendation and under the supervision of a physician
**12**	Omega-3	Biopharma	fish oil	Norway	EPA 0.0355, DHA 0.137	Vitamin E 5mg, Vitamin D 10µg	1 cps 2 times	capsules, 120, 84 g	EPA and DHA contribute to the normal function of the heart. Vitamin D contributes to the normal function of the immune system. Folate contributes to normal tissue formation in pregnant women	A nutritional supplement should not be a substitute for a balanced and varied diet and a healthy lifestyle. Keep out of reach of children	The recommended daily dose should not be exceeded. Not recommended for children under 3 years old
**13**	Trippel Omega-3 kvinne	Biopharma	fish oil, evening primrose oil	Norway	EPA 0.151, DHA 0.1015, GLA 0.005	Vitamin E 6 mg Vitamin D 10 µg, Vitamin K 12.5 µg, Folic acid 100 µg	2	capsules, 120, 115 g	EPA and DHA contribute to the normal function of the heart. Vitamin D contributes to the normal function of the immune system	A nutritional supplement should not be a substitute for a balanced and varied diet and a healthy lifestyle. Keep out of reach of children	The recommended daily dose should not be exceeded. Not recommended for children under 3 years old
**14**	Omega-3 fish oil	Vitalikum	fish oil	Hungary	EPA 0.18, DHA 0.12	/	1	capsules, 100, 134 g	EPA and DHA contribute to the normal function of the heart (a beneficial effect is achieved with a daily intake of 250 mg of EPA and DHA)	A nutritional supplement should not be a substitute for a balanced and varied diet. Keep out of reach of children	Recommended daily doses must not be exceeded. Before using this product, you should consult a doctor or pharmacist. It is not recommended for pregnant and lactating women, as well as for people sensitive to any of the ingredients

**15**	Trippel Omega-3	Biopharma	fish oil	Norway	EPA 0.155, DHA 0.125	Vitamin E 5 mg, Vitamin D 8 µg, Vitamin A 125 µg	2	softgel capsules, 144	EPA and DHA contribute to normal heart function and normal vision (a beneficial effect is achieved with a daily intake of 250 mg of DHA AND EPA). Vitamin D contributes to the normal function of the immune system	A nutritional supplement should not be a substitute for a balanced and varied diet	The recommended daily dose should not be exceeded. Not recommended for children under 3 years old
**16**	Omega 3+ Q10	Mintmedic	fish oil	/	EPA 0.18, DHA 0.12	coenzyme Q10 30mg	1	capsules, 30	DHA contributes to maintaining normal triglyceride levels, as well as normal brain development. EPA and DHA contribute to normal heart function, maintenance of normal blood pressure and maintenance of normal cholesterol and triglyceride levels	A nutritional supplement should not be a substitute for a balanced and varied diet. Keep out of reach of children	The recommended daily amounts must not be exceeded. The product should not be taken by people who are hypersensitive to any of the ingredients of the product. The product is not intended for children under 6 years of age. Excessive use can cause a laxative effect
**17**	Natural Omega-3	Natural Welth	fish oil	USA	/	/	1	softgel capsules,100	EPA and DHA contribute to normal heart function and normal vision (a beneficial effect is achieved with a daily intake of 250 mg of DHA AND EPA). Vitamin D contributes to the normal function of the immune system	A nutritional supplement should not be a substitute for a balanced and varied diet. Keep out of reach of children. Do not use if the protective film is damaged or removed	The recommended daily dose should not be exceeded. In case of pregnancy, breastfeeding, use of medicines, planned medical interventions or the existence of a medical condition, it is necessary to consult a doctor before use. In case of any unwanted reaction, discontinue use and consult a doctor
**18**	Omega 3will	Goodwill Pharma	fish oil	Serbia	EPA 0.18, DHA 0.12	Vitamin E 5 mg	1	softgel capsules,60, 82.5 g	EPA and DHA contribute to the normal function of the heart (a beneficial effect is achieved with a daily intake of 250 mg of EPA and DHA.). DHA contributes to the maintenance of normal brain function and maintenance of normal	Supplement is not a substitute for a balanced, varied diet and leading a healthy lifestyle. Keep out of reach of children	Recommended daily doses must not be exceeded. People who are on therapy with antiplatelet and anticoagulant drugs should consult a doctor before use. It is not recommended for people sensitive to any ingredient of the product. The product can be used by pregnant and

									vision (a beneficial effect will be achieved with a daily intake of 250 mg of DHA). Vitamin E contributes to the protection of cells from oxidative stress		lactating women after consultation with a doctor
**19**	Omega3	Together Health	algae oil	United Kingdom	EPA 0.123, DHA 0.207	/	1	softgel capsules, 30	The image of *the heart* emphasizes health	A nutritional supplement should not be a substitute for a balanced and varied diet	Recommended daily doses must not be exceeded. Do not use if the packaging is damaged
**20**	Omega vite	Max Medica	fish oil	Serbia	EPA 0.18, DHA 0.12	Vitamin E 5.4 mg, Zinc 10 mg	1 to 2	softgel capsules, 60	EPA and DHA contribute to the normal function of the heart (a beneficial effect is achieved with a daily intake of 250 mg of EPA and DHA). Vitamin E and zinc contribute to the protection of cells from oxidative stress. Zinc contributes to the normal metabolism of fatty acids, carbohydrates, vitamin A, and macronutrients. Zinc contributes to the maintenance of normal bones, skin, nails and vision. Zinc contributes to normal protein synthesis, immune system function as well as normal DNA synthesis and plays a role in the process of cell division	Nutritional supplements cannot be used as a substitute for a varied and balanced diet and leading a healthy lifestyle. Keep out of reach of children	Recommended daily doses should not be exceeded. The product should not be taken by people who are sensitive to any ingredient. The product is not intended for children under 4 years of age. Pregnant and lactating women should consult a doctor before taking. People who are on warfarin therapy or suffer from haemophilia, as well as diabetics, are recommended to consult a doctor before taking sea fish. It is not recommended to take marine fish for people who are preparing for surgical intervention

**21**	Omega 3 + vitamin E	Solevita	fish oil	Serbia	EPA 0.18, DHA 0.12	Vitamin E 5 mg	1	softgel capsules, 60, 82 g	EPA and DHA contribute to the normal function of the heart (a beneficial effect is achieved with a daily intake of 250 mg of EPA and DHA). Vitamin E contributes to the protection of cells from oxidative stress	Nutritional supplements cannot be used as a substitute for a varied and balanced diet. Keep out of reach of children	Recommended daily doses should not be exceeded. The product should not be taken by people who are hypersensitive to any of the product's ingredients. The product is not intended for pregnant women, lactating women and those under 18 years of age. It is not recommended for people on anticoagulant therapy and it is not used two weeks before planned surgical procedures. People who take aspirin, nonsteroidal anti-inflammatory drugs, as well as people who suffer from haemophilia and diabetes, should consult their doctor before taking supplements with fish oil. Excessive use can cause a laxative effect
**22**	Fish pearls with vitamins A and D	Lifeline	fish oil	Bosnia and Herzegovina (Belgium)	EPA 0.054, DHA 0.036	Vitamin A 400 µg, Vitamin D 10 µg	1	capsules, 30, 5.55 g	Vitamins A and D contribute to the normal function of the immune system. Vitamin A contributes to the maintenance of the normal mucosa of the membrane. Vitamin D contributes to the maintenance of normal bones and teeth	Nutritional supplements cannot be used as a substitute for a varied and balanced diet. Keep out of reach of children	Recommended daily doses should not be exceeded. The product should not be taken by people who are hypersensitive to any of the ingredients of the product. Pregnant and nursing women should consult a doctor/pharmacist before using the product. If there is already a prescribed therapy, consult a doctor/pharmacist before use. Caution is required in people on anticoagulant therapy, diabetics and people suffering from haemophilia. The product is not intended for children under 3 years of age

**23**	Omega-3 fish oil	Now Foods	fish oil	USA (Morocco)	EPA 0.18, DHA 0.12	/	2	softgel capsules, 200	The use of EPA and DHA may reduce the risk of coronary heart disease. EPA and DHA support heart health	Nutritional supplements are not a substitute for a balanced diet and a healthy lifestyle. The supplement is not intended for children and adults under 18 years of age. Keep out of reach of children	Recommended daily doses must not be exceeded. Pregnant/nursing women, people using therapy or having a medical condition should consult a doctor before use
**24**	Epobalans primrose oil + vitamin E	Strong Nature	evening primrose oil	Spain	GLA 0.1	Vitamin E 5 mg	2 to 3	capsules,30, 42.2g	Vitamin E contributes to the protection of cells from oxidative stress	Supplement is not a substitute for a varied diet and a healthy lifestyle. Keep out of reach of children	The recommended daily dose must not be exceeded. It is not recommended for children, pregnant women, nursing mothers, as well as for people who are hypersensitive to any of the product's ingredients. Application of the supplement should be stopped before any surgical intervention
**25**	Omega 3-6-9 fish-flax-borage	Natural Welth	fish oil, linseed oil, borage oil	USA	EPA 28.5%, DHA 17%, ALA 45%	Vitamin E 3.35 mg	1 to 3	softgel capsules, 60, 103.92 g	EPA and DHA contribute to the normal function of the heart (a beneficial effect is achieved with a daily intake of 250 mg of EPA and DHA). ALA contributes to maintaining a normal level of cholesterol in the blood, this effect is achieved with a daily ounce of 2 g of ALA through food and dietary supplements	Supplement is not a substitute for a varied diet and a healthy lifestyle. Keep out of reach of children. Do not use if the protective film is damaged or removed	Recommended daily doses must not be exceeded. Pregnant/nursing women, people using therapy, including blood thinners, planning surgery or having a medical condition should consult a doctor before use. In case of some unwanted reactions, discontinue use

**26**	Omega 3	Solgar	fish oil	USA	EPA 0.16, DHA 0.1	/	1	capsules, 60, 87 g	EPA and DHA contribute to the normal function of the heart (a beneficial effect is achieved with a daily intake of 250 mg of EPA and DHA)	Nutritional supplements cannot be used as a substitute for a varied and balanced diet. Keep out of reach of children	The recommended daily dose must not be exceeded. The product should not be taken by people who are hypersensitive to any of the ingredients. The product is not intended for pregnant women, lactating women and those under 18 years of age, except on the advice of a doctor. Supplements based on fish oil can lower blood pressure and prolong coagulation, so they are not recommended for people on anticoagulant therapy and should not be used two weeks before planned surgical procedures. People who take aspirin, anti-inflammatory drugs, as well as people who suffer from haemophilia and diabetes should consult their doctor before taking supplements with fish oil
**27**	Omega 3	Synergy	fish oil	USA	EPA 0.18, DHA 0.12	Vitamin E 3.35 mg	1	softgel capsules, 30	EPA and DHA contribute to normal heart function, maintenance of normal blood pressure and maintenance of normal cholesterol and triglyceride levels	Keep out of reach of children	In case of using any medicine, pregnancy or breastfeeding, consult a doctor
**28**	0mega junior	Pharmalife	blackcurrant and soybean seed oil	Italy	ALA 0.294	Vitamin E 3 mg, Vitamin B6 1.5 mg	infants 5 drops/day; infants up to 1 year 30 drops twice a day; >1 year and adults 30 drops 3 times a day	liquid, 30 ml, 27 g	It contributes to the support of the physiological health of infants and young children	Nutritional supplements are not a substitute for a varied diet and a healthy lifestyle. Keep out of the reach of small children	The recommended daily dose must not be exceeded. The product should not be taken by people who are hypersensitive to any of the ingredients

**29**	Evening primrose oil	Lilly Pharmacy	evening primrose oil	Serbia	GLA 0.1	Vitamin E 13.42 mg	1	capsules, 30, 42.6 g	Vitamin E contributes to the protection of cells from oxidative stress. Evening primrose contributes to skin health, hormonal health and regulation of metabolic functions	Nutritional supplements are not a substitute for a varied and balanced diet. Keep out of reach of children	Supplement should not be taken by people who are hypersensitive to any of the product's ingredients. This supplement is not intended for pregnant women, lactating women and those under 18 years of age. It is not recommended for people suffering from epilepsy, schizophrenia and people on therapy with drugs from the group of aliphatic phenyl tiazides. Excessive use can cause a laxative effect
**30**	Orthomol nathal	Orthomol	fish oil	Germany	EPA 0.02, DHA 0.15	Vitamin E 36 mg, Ca 400 mg	2	capsules, 30	Docosahexaenoic acid (DHA) maternal intake contributes to the normal development of the brain and eye of the foetus and breastfed infants (a beneficial effect is achieved with an intake of 200 mg of DHA per day in addition to the recommended daily intake for omega-3 fatty acids for adults, i.e. 250 mg of DHA and EPA). Calcium is necessary for the maintenance of normal bones, calcium participates in the process of cell division	Nutritional supplements are not a substitute for a varied and balanced diet and a healthy lifestyle. Keep out of reach of children	Recommended daily doses must not be exceeded. Contains iodine. If you suffer from hyperfunction of the thyroid gland, consult your doctor before taking this supplement Contains readily available carbohydrates. This supplement should be used only with careful control of the metabolism of disturbances in glucose tolerance. -this refers to the part that is not the omega capsule
**31**	Wellness pack woman	Oriflame	fish oil	Sweden	EPA 0.075, DHA 0.05	/	2	capsules, 63	The name *WELLNESS* emphasizes health	Nutritional supplements are not a substitute for a balanced diet. Keep out of reach of children. Do not use if the protective film is damaged or removed	The recommended daily dose must not be exceeded. Consult a doctor before use if you have certain medical conditions, if you are pregnant or planning to become pregnant

**32**	Welness kids omega 3	Oriflame	fish oil	United Kingdom	EPA 0.4, DHA 0.5	Vitamin E 5.5 mg	5 ml	liquid, 105 ml	The image of *the sun* emphasizes health	Intended for children older than 3 years	/
**33**	Omega 3 Seefisch ol 1000 Doppel herz	Quiesser Pharma	fish oil	Germany	EPA 0.18, DHA 0.12	Vitamin E 3.35 mg	1	capsules, 80, 107.8g	EPA and DHA contribute to the normal function of the heart (a beneficial effect is achieved with a daily intake of 250 mg of EPA and DHA). Vitamin E contributes to the protection of cells from oxidative stress	Nutritional supplements cannot be used as a substitute for a varied and balanced diet. Keep out of reach of children	Recommended daily doses should not be exceeded. The product should not be taken by people who are hypersensitive to any of the ingredients. The product is not intended for children under 12 years of age. Supplements based on fish oil can affect the lowering of blood pressure and the prolongation of coagulation, so they are not recommended for people on anticoagulant therapy and should not be used two weeks before planned surgical procedures. People who take aspirin, non-steroid anti-inflammatory drugs, as well as people who suffer from haemophilia and diabetes, should consult their doctor before taking supplements with fish oil. Excessive use can cause a laxative effect
**34**	Omega Defend	PharmaSw iss	fish oil	Poland	EPA 0.235, DHA 0.191	/	2	capsules, 60, 64.98 g	EPA and DHA contribute to the normal function of the heart (a beneficial effect is achieved with a daily intake of 250 mg of EPA and DHA)	Nutritional supplements are not a substitute for a varied and balanced diet. Exercising healthy lifestyle habits as well as a varied diet are prerequisites for good health	The recommended daily dose must not be exceeded
**35**	Omega 3	Natura Aucta	fish oil	Bulgaria	EPA 0.18, DHA 0.12	Vitamin E 10 mg	1 to 2	softgel capsules,60 ,84 g	Omega-3 fatty acids have a favourable effect on the health of the cardiovascular system and on the level of triglycerides, total and	Nutritional supplements cannot be used as a substitute for a varied and balanced diet. Not	The recommended daily dose must not be exceeded. This product should be used with caution in people who are on antiplatelet and anticoagulant therapy

									LDL cholesterol. Vitamin E has a positive effect on cell metabolism and cell protection	recommended for use in children	
**36**	Super EPA fish oil 1000mg SGC	M.E.V. Feller	fish oil	United Kingdom	EPA 0.33, DHA 0.22	/	1	softgel capsules, 60, 84.12 g	EPA and DHA contribute to the normal function of the heart (a beneficial effect is achieved with a daily intake of 250 mg of EPA and DHA). DHA maternal intake contributes to the normal development of the eye of the foetus and breastfed infants (a beneficial effect is achieved with a minimum intake of 200 mg of DHA per day)	Nutritional supplements cannot be used as a substitute for a varied and balanced diet.	Recommended daily doses should not be exceeded. The product should not be taken by people who are hypersensitive to any of the ingredients. The product is not intended for children under 6 years of age. Supplements based on fish oil can lower blood pressure and prolong coagulation, so it is not recommended for people on anticoagulant therapy and should not be used two weeks before planned surgical procedures. People who take aspirin, nonsteroidal anti-inflammatory drugs, as well as people who suffer from haemophilia and diabetes, should consult their doctor before taking supplements with fish oil.
**37**	Omega 3 classic	Dr Max Pharma	fish oil	Czech Republic	EPA 0.16, DHA 0.1	Vitamin E 3.35 mg	1 to 3	capsules, 90, 120.6 g	EPA and DHA contribute to the normal function of the heart (a beneficial effect is achieved with a daily intake of 250 mg of EPA and DHA.). DHA contributes to the maintenance of normal brain function (a beneficial effect will be achieved with a daily intake of 250 mg of DHA). Vitamin E contributes to the protection of cells from oxidative stress	Nutritional supplements are not a substitute for a varied and balanced diet and a healthy lifestyle. Keep out of reach of children.	Recommended daily doses must not be exceeded. It is not recommended for people sensitive to any ingredient of the supplement It is not intended for children under 3 years of age.

**38**	Omega 3 premium	Dr Max Pharma	fish oil	Czech Republic	EPA 0.225, DHA 0.17	/	1 to 2	capsules, 90, 61.2 g	EPA and DHA contribute to the normal function of the heart (a beneficial effect is achieved with a daily intake of 250 mg of EPA and DHA.). DHA contributes to the maintenance of normal brain function and maintenance of normal vision (a beneficial effect will be achieved with a daily intake of 250 mg of DHA)	Nutritional supplements are not a substitute for a varied and balanced diet and a healthy lifestyle. Keep out of reach of children.	Recommended daily doses must not be exceeded. It is not recommended for people sensitive to any ingredient of the supplement It is not intended for children under 3 years of age.
**39**	Omega 3 enriched formula	Adrien Gagnon	fish oil	Canada	EPA 0.3, DHA 0.2	/	adults 1 to 3, pregnant and lactating women 1	softgel capsules, 80, 112.2 g	DHA contributes to normal brain function and normal vision (a beneficial effect is achieved with a minimum intake of 250 mg of DHA per day). EPA and DHA contribute to the normal function of the heart (a beneficial effect is achieved with a minimum intake of 250 mg of EPA and DHA per day). The maternal intake contributes to the normal development of the brain and vision of the foetus and infants (a beneficial effect is achieved with a minimum intake of 200 mg of DHA per day)	Nutritional supplements cannot be used as a substitute for a varied and balanced diet. Keep out of reach of children	The recommended daily dose must not be exceeded. Products based on fish oil can lower blood pressure and prolong coagulation, so they are not recommended for people on anticoagulant therapy and should not be used two weeks before planned surgical procedures. People taking aspirin, non-steroidal anti-inflammatory drugs, as well as people suffering from haemophilia and diabetes should consult a doctor before taking supplements with fish oil. The product is not intended for children under 6 years of age
**40**	Wild salmon and fish oils	Jamieson Laboratori es	fish oil	Canada	EPA 0.18, DHA 0.12	/	adults and children over 12 years 1-3	softgel capsules, 90, 146.43 g	EPA and DHA contribute to the normal function of the heart (a beneficial effect is achieved with a minimum intake of 250 mg of EPA and DHA per day). DHA contributes to the maintenance of normal brain function and normal	Nutritional supplements cannot be used as a substitute for a varied and balanced diet. It is important to follow a balanced and varied diet and a healthy lifestyle.	The recommended daily dose must not be exceeded. The product must not be used by people who are hypersensitive to any ingredient of the supplement

									vision. The maternal intake contributes to the normal development of the brain and vision of the foetus and infants (a beneficial effect is achieved with a minimum intake of 200 mg of DHA per day with the recommended daily intake of omega-3 fatty acids for adults, 250mg of EPA and DHA)	Keep out of reach of children. Capsules must not be chewed.	
**41**	Ginko omega	Dietpharm	fish oil	Croatia (France)	DHA 0.25	dry Ginkgo leaf extract 60 mg, Niacin 10 mg, Pantothenic acid 6 mg, Vitamin B6 2 mg, Vitamin B2 1.6 mg, Vitamin B1 1.4 g, Folic acid 200 µg, Vitamin B12 1 µg	adults 1, for 2 months, then take a break for 1 month	capsules, 30, 28.2 g	DHA contributes to maintenance of normal brain function (a beneficial effect is achieved with a daily intake of 250 mg of DHA). Pantothenic acid contributes to normal mental abilities. Ginkgo leaf extract contributes to maintaining normal circulation and memory	Nutritional supplements cannot be used as a substitute for a varied and balanced diet. It is important to follow a balanced diet and a healthy lifestyle. Keep out of reach of children	The recommended daily dose must not be exceeded. The product should not be taken by people who are hypersensitive to any of the ingredients. It is not intended for people who take drugs to prevent blood clotting, lower blood sugar levels, drugs against high blood pressure, convulsions and depression. Taking supplements based on ginkgo should be stopped at least 36 hours before any planned surgical intervention. Simultaneous use of supplements based on ginkgo and oral anticoagulants, non-steroidal anti-inflammatory drugs, as well as aspirin can lead to increased bleeding. People who have an increased risk of intracranial bleeding should avoid ginkgo supplements. The simultaneous use of supplements based on Ginkgo biloba with garlic, 5-hydroxytryptophan and salbal palm is not recommended. The product is not intended for

											pregnant women, lactating women and those under 18 years of age
**42**	O3 omega 3 plus vitamin E	Immuno Systems	fish oil	Serbia	EPA 0.18, DHA 0.12	Vitamin E 5 mg	1 to 2	capsules, 30, 41.25 g	Cardioprotective effect. Taking DHA contributes to the normal development of infants up to 12 months . Vitamin E contributes to the protection of cells from oxidative stress	Nutritional supplements are not a substitute for a varied and balanced diet. Keep out of reach of children	Recommended daily doses must not be exceeded. Not recommended for use in children. This product should be used with caution in people who are on antiplatelet and anticoagulant therapy. In case of hypersensitivity to any of the ingredients, it is necessary to stop taking it and consult a doctor
**43**	Omega max kids	MaxMedica	fish oil	Serbia	EPA 0.147, DHA 0.097	/	children 6-12 months 1 ml, 1-3 years 1.5 ml, 4-18 years 2.5 ml, adults 5 ml	liquid, 100 ml	DHA contributes to the maintenance of normal brain function and maintenance of normal vision (a beneficial effect is achieved with a daily intake of 250 mg of DHA). EPA and DHA contribute to the normal function of the heart (a beneficial effect is achieved with 250 mg of EPA and DHA/day). DHA intake during pregnancy and breastfeeding contributes to normal eye development in foetuses and breast-fed infants (a beneficial effect is achieved by taking 200 mg of DHA/day with the recommended intake of omega-3 fatty acids for adults, 250 mg of DHA and EPA). DHA intakes contributes to the normal development of infants up to 12 months (a beneficial	Nutritional supplements are not a substitute for a varied diet and a healthy lifestyle. Keep out of reach of children. Consult a doctor or pharmacist before use	The recommended daily dose must not be exceeded. The product must not be used by persons allergic to any of the ingredients of the product. People using anticoagulant therapy, antiplatelet therapy, oral contraceptives, orlistat should consult their doctor before starting to use omega-3 oil. People under the age of 18, pregnant and lactating women should use this product only on the recommendation and under the supervision of a doctor

									effect is achieved by 100 mg of DHA/day)		
**44**	Omega 3 extreme	Ostrovit	fish oil	Poland	EPA 0.5, DHA 0.25	/	1	capsules, 90, 117 g	EPA and DHA contribute to the normal function of the heart (a beneficial effect is achieved with a daily intake of 250 mg of EPA and DHA)	Nutritional supplements are not a substitute for a balanced diet. A balanced and healthy diet is recommended. Keep out of reach of children	The recommended daily dose must not be exceeded. Do not use in case of allergy to any ingredient. It is not recommended for children, pregnant and lactating women, as well as for people who are hypersensitive to any of the ingredients. People with haemophilia and those taking warfarin should be careful when using fish oil supplements due to the possible antithrombotic effect of fish oil. Fish oil supplements should be discontinued prior to any surgical procedure. People with any chronic disease should consult a doctor before use
**45**	Omega 3 gold	Maxler	fish oil	Germany	EPA 0.18, DHA 0.12	Vitamin E 3 mg	1 cps 1-3 times	capsules, 120, 161 g	The name *GOLD* emphasizes the potential benefit	Nutritional supplements are not a substitute for a varied and balanced diet and a healthy lifestyle. Keep out of reach of children	The recommended daily dose must not be exceeded. Pregnant and lactating women should not use doses higher than recommended. The product should not be used by people who are hypersensitive to any of the ingredients. People who have a diagnosed disease and/or use medications should consult a doctor or pharmacist before using this product. People with blood coagulation disorders and disorders with a tendency to bleeding should use the product with caution and with an agreement with a doctor. The use of the product should

											be stopped one month before the surgical intervention
**46**	Nutriversum Omega 3	Bedeco KFT	fish oil	EU	EPA 0.18, DHA 0.12	/	1 cps 1-2 times	softgel capsules, 90, 123 g	EPA and DHA contribute to the normal function of the heart (a beneficial effect is achieved with a daily intake of 250 mg of EPA and DHA.). DHA contributes to the maintenance of normal brain function and maintenance of normal vision (a beneficial effect will be achieved with a daily intake of 250 mg of DHA)	Nutritional supplements cannot be used as a substitute for a varied and balanced diet and a healthy lifestyle. Keep out of reach of children. Consult a doctor or pharmacist before use	The recommended daily dose must not be exceeded. The product is not recommended for people who are sensitive to some of the ingredients. Pregnant and lactating women should consult a doctor before taking this product. People with haemophilia and those taking warfarin should be careful when using fish oil supplements due to the possible antithrombotic effect of fish oil. Fish oil supplementation should be discontinued prior to any operative procedure
**47**	BABYTOL DHA omega	4U Pharma	algae oil	Switzerland (Poland)	DHA 0.1	/	1	twist-off capsules 30, 14,1 g	Docosahexaenoic acid (DHA) intake contributes to the normal visual development of infants up to 12 months of age (a beneficial effect is achieved with a daily intake of 100 mg of DHA)	Supplement contains ingredients of natural origin, which can cause clouding of the contents of the capsule, especially when stored at lower temperatures. This phenomenon does not affect the quality of the product. A dietary supplement is not a substitute or substitute for a balanced diet. It is important to follow a balanced and varied diet and a healthy lifestyle. Do not put the whole capsule in your mouth. squeeze out only its contents	Pregnant and lactating women should consult their doctor or pharmacist before using this product. The product should not be taken by people who are hypersensitive to any of the ingredients

**48**	BABYTOL D3+ DHA omega	4U Pharma	algae oil	Switzerland (Poland)	DHA 0.1	Vitamin D 10 µg (400 IU)	1	twist-off capsules 30, 14,1 g	Docosahexaenoic acid (DHA) intake contributes to the normal visual development of infants up to 12 months of age (a beneficial effect is achieved with a daily intake of 100 mg of DHA). Vitamin D is necessary for bone growth and development in children	Supplement contains ingredients of natural origin, which can cause clouding of the contents of the capsule, especially when stored at lower temperatures. This phenomenon does not affect the quality of the product. A dietary supplement is not a substitute or substitute for a balanced diet. It is important to follow a balanced and varied diet and a healthy lifestyle. Do not put the whole capsule in your mouth. squeeze out only its contents	Pregnant and lactating women should consult their doctor or pharmacist before using this product. The product should not be taken by people who are hypersensitive to any of the ingredients
**49**	NOVALAC prenatalne kapsule	Medis	fish oil	Austria (Spain)	DHA 0.2	Folic acid 600 µg, Fe 14 mg, Mg 56 mg, Zn 1 g, I 13.4 mg, Se 4.5 mg, B vitamins (B1 1.4 mg, B2 1.4 mg, Niacin 18 mg, Pantothenic acid 6 mg, B6 0.2 mg, B12 0.1 µg, Folic acid 400 µg) Vit C 3.6 g, Vit D 0.9 mg, Vit E 1.3 g	1	capsules, 30, 33.6 g	DHA maternal intake contributes to the normal development of the brain and eye of the foetus and breastfed infants (a beneficial effect is achieved with an intake of 200 mg of DHA per day in addition to the recommended daily intake for omega-3 fatty acids for adults, i.e. 250 mg of DHA and EPA). Low maternal folate is a risk factor for developing neural tube defects. In women in the reproductive period, a beneficial effect is achieved with an additional daily intake of at least 400 µg	A nutritional supplement is not a substitute for a balanced and varied diet and a healthy lifestyle. Keep out of reach of children	The recommended daily dose must not be exceeded. People who take medicines should consult a doctor or pharmacist before taking them

**50**	Omega 3	Dietpharm	fish oil	Croatia (Greece)	EPA 0.18, DHA 0.12	/	1 to 2	capsules, 50, 70,5 g	EPA and DHA contribute to the normal function of the heart (a beneficial effect is achieved with a daily intake of 250 mg of EPA and DHA)	A nutritional supplement is not a substitute or substitute for a balanced diet. It is important to follow a balanced and varied diet and a healthy lifestyle. Keep out of reach of children	The recommended daily dose must not be exceeded. People who take medications that affect blood clotting should consult a doctor before using this product
**51**	OMEGA + vitamin E	Pharmamed	fish oil	Bosnia and Herzegovina	EPA 0.16, DHA 0.1	Vitamin E 1.2 mg	1 do 2	capsules, 30	EPA and DHA contribute to the normal function of the heart (a beneficial effect is achieved with a daily intake of 250 mg of EPA and DHA)	A nutritional supplement is not a substitute for a balanced diet. Keep out of reach of children	Supplement should not be taken by people who are hypersensitive to any of the ingredients. People who drink medicines that can affect blood clotting should consult a doctor before taking this supplement. Pregnant and lactating women should consult a doctor before using all medicines and food supplements, including this product
**52**	OMEGA 3 1000mg + 12mg Vitamin E	PharmaVital	fish oil	Germany	EPA 0.18, DHA 0.12	Vitamin E 12 mg, Vitamin D 10 µg	1	capsules, 100, 137.7 g	For the protection of the heart and blood vessels. Vitamin E contributes to the protection of cells from oxidative stress	A nutritional supplement is not a substitute for a balanced and varied diet and a healthy lifestyle. Keep out of reach of children	The recommended daily dose must not be exceeded
**53**	Omega 3 Leinol 1.000	Mivolis	linseed oil	Germany	ALA 0.508	Vitamin E 12 mg, Vitamin B6 4.2 mg, Folic acid 600 µg, Vitamin B12 7.5 µg	1	capsules, 30, 46.6 g	Folic acid, vitamin B6 and B12 contribute to the normal metabolism of homocysteine. Vitamin E contributes to the protection of cells from oxidative stress	A nutritional supplement is not a substitute or substitute for a balanced diet. Keep out of reach of children	The recommended daily dose must not be exceeded

**54**	SAVE OIL PLUS	Inpharm	shrimp oil	Serbia (USA)	/	Astaxanthin 0.05 mg, Coenzyme Q10 51.5 mg	1	capsules, 30, 25.14 g	The image of *the cross* emphasizes the health of the cardiovascular system	Nutritional supplements are not a substitute for a varied and balanced diet and a healthy lifestyle. Keep out of reach of children	The recommended daily dose must not be exceeded. This product is not intended for pregnant women, lactating women, people under 18 years of age, people using anti-clotting drugs, haemophiliacs, as well as people allergic to soy, fish and crustaceans. Stop using the supplement 7 days before surgery. In case of hypersensitivity, stop taking the product immediately and consult a doctor
**55**	Multivita omega 3	Fidifarm	fish oil	Croatia (Greece)	EPA 0.18, DHA 0.12	/	1 cps 2-3 times	capsules, 30, 42 g	EPA and DHA contribute to the normal function of the heart (a beneficial effect is achieved with a daily intake of 250 mg of EPA and DHA)	Nutritional supplements cannot be used as a substitute for a varied and balanced diet. It is important to follow a balanced diet and a healthy lifestyle. Keep out of reach of children	The recommended daily dose must not be exceeded. People who use drugs that affect blood clotting should consult a doctor before taking this product. The product should not be used by people who are hypersensitive to any ingredient
**56**	Fish oil	Galenfarm	fish oil	Bosnia and Herzegovina	/	Vitamin A 112000 IU/100g. Vitamin D 11800IU/100g	children 1-3 years 1.25 ml; children 4-7 years 1.75 ml; children 7-14 years 2.5 ml; children over 14 and adults 4 ml	liquid, 100 ml	Omega-3 fatty acids have a beneficial effect on the functioning of the cardiovascular system, brain, immunity and improvement of dry skin. Vitamin D contributes to the normal absorption and utilization of calcium and phosphorus. Vitamin A contributes to the maintenance of normal vision. Vitamin A contributes to the normal function of the immune system	A nutritional supplement is not a substitute or substitute for a balanced diet. Keep out of reach of children	The recommended daily dose must not be exceeded. For use by pregnant and lactating women, it is necessary to consult a doctor. It should not be used in case of hypersensitivity to any of the ingredients. Simultaneous use with other supplements containing vitamins A and D is not recommended

**57**	Fish oil Jecoris oleum	MaxiVita	fish oil	Bosnia and Herzegovina	/	/	10 ml, during 10 days	liquid, 100 ml	Fish oil has a beneficial effect on the skin and face, helps with acne-prone skin and psoriasis. Improves hair growth, regulates body weight. Protects the heart, brain and joint stiffness. It is considered a good agent against nervous diseases, depression, improves vision. It is an excellent anti-aging agent and even anti-cancer because it regenerates the liver	/	/
**58**	ORTHOMOL Cardio	Orthomol	fish oil	Germany	EPA 0.15, DHA 0.083	/	2	capsules, 90	EPA and DHA contribute to the normal function of the heart (a beneficial effect is achieved with a daily intake of 250 mg of EPA and DHA)	Nutritional supplements are not a substitute for a varied and balanced diet and a healthy lifestyle. Keep out of reach of children	The recommended daily dose must not be exceeded
**59**	ORTHOMOL I-care granulea30	Orthomol	fish oil	Germany	EPA 0.097, DHA 0.65	/	2	capsules, 60, 51 g	For dietary treatment in cancer patients	Nutritional supplements are not a substitute for a varied and balanced diet and a healthy lifestyle. Keep out of reach of children	The recommended daily dose must not be exceeded
**60**	ORTHOMOL Arthroplus	Orthomol	fish oil	Germany	EPA 0.25, DHA 0.055	/	2	capsules, 60, 42 g	For the dietary treatment of osteoarthritic changes in the joints	Supplement is not for parenteral use. It is not suitable for use as the sole source of food for the people for whom it is intended.	Use only under the supervision of a doctor. It must not be used during pregnancy and breastfeeding, nor must it be given to children. If drugs that affect blood coagulation are used, coagulation parameters should be checked at regular intervals, especially at the beginning and end of treatment. People allergic to any component included in the composition of the supplement should not use it

**61**	ORTHO MOL vital	Orthomol	fish oil	Germany	EPA 0.3, DHA 0.2	Folic acid 400 µg	2	capsules, 60, 45 g	EPA and DHA contribute to the normal function of the heart (a beneficial effect is achieved with a daily intake of 250 mg of EPA and DHA)	Nutritional supplements are not a substitute for a varied and balanced diet and a healthy lifestyle. Keep out of reach of children	The recommended daily dose must not be exceeded.
**62**	ORTHO MOL vital M	Orthomol	fish oil	Germany	EPA 0.15, DHA 0.1	/	2	capsules, 60, 54 g	EPA and DHA contribute to the normal function of the heart (a beneficial effect is achieved with a daily intake of 250 mg of EPA and DHA)	Nutritional supplements are not a substitute for a varied and balanced diet and a healthy lifestyle. Keep out of reach of children	The recommended daily dose must not be exceeded
**63**	ORTHO MOL vital F	Orthomol	fish oil	Germany	EPA 0.15, DHA 0.1	/	1	capsules, 30, 33 g	EPA and DHA contribute to the normal function of the heart (a beneficial effect is achieved with a daily intake of 250 mg of EPA and DHA)	Nutritional supplements are not a substitute for a varied and balanced diet and a healthy lifestyle. Keep out of reach of children	The recommended daily dose must not be exceeded
**64**	ORTHO MOL junior Omega plus	Orthomol	fish oil	Germany	EPA 0.017, DHA 0.1, GLA 0.005	Fe 0.77 mg, Mg 33mg, Zn 3.5 mg, Vit E 3.3 mg, Vit C 17 mg, Biotin 5 µg, Vit B1 0.2 mg, Vit B2 0.23 mg, Vit B6 1 mg, Pantothenic acid 1.3 mg, Folic acid 33 µg	children 3 caramels	caramels, 90, 432 g	Fe contributes to the normal cognitive development of children	Nutritional supplements are not a substitute for a varied and balanced diet and a healthy lifestyle. Keep out of reach of children	The recommended daily dose must not be exceeded. It is intended for children aged 4 and older

**65**	Cerebronal	Biocinalis	fish oil	EU	DHA 0.2325	Vit D 10 µg, Astaxanthin 3 mg, Niacin 5 mg, Pantothenic acid 5 mg, Thiamin 2 mg, Vit B6 2 mg, Riboflavin 2 mg, Folic acid 400µg, Vit B12 10µg	1	capsules, 45, 27.45 g	DHA contributes to maintenance of normal brain function. Vitamin D contributes to the normal function of the immune system. Vitamins of the B group contribute to the normal function of the nervous system, normal mental work, etc	Nutritional supplements cannot be used as a substitute for a varied and balanced diet. It is important to follow a balanced diet and a healthy lifestyle. Keep out of reach of children	The recommended daily dose must not be exceeded. In case of pregnancy, breastfeeding, hypersensitivity or allergy to any of the ingredients of the product, consult a doctor before use. This product must not be used by children and adolescents under the age of 14
**66**	ConCordix Smart Chews algae oil	Vitux As	algae oil	Norway	/	/	1 to 2	candies	DHA from algae supports brain development and function, aids normal cognitive function at all stages of life, promotes healthy visual function and heart health	Nutritional supplements cannot be used as a substitute for a varied and balanced diet and a healthy lifestyle. Keep out of reach of children	The recommended daily dose must not be exceeded. It can have a laxative effect if used in excess
**67**	ConCordix Smart Chews omega double boost	Vitux As	fish oil	Norway	EPA 0.32, DHA 0.24	/	1 to 2	candies	Omega-3 fatty acids support brain function, aiding normal cognitive function throughout all life stages	Nutritional supplements cannot be used as a substitute for a varied and balanced diet and a healthy lifestyle. Keep out of reach of children	The recommended daily dose must not be exceeded. It can have a laxative effect if used in excess
**68**	Odor-free vegan omega-3	Bonchabio	algae oil	Taiwan	/	/	1	liquid	It supports brain function, vision and heart health	/	/
**69**	Magic pow(d)er mix protein blend	Henry Lamote Oils	hemp, flax, spirulina oil	Germany	/	/	15 g	powder, 15 g	The name *POW(d)ER* emphasizes the potential benefit	/	/

**70**	MOLLERS Omega-3 Citron	Orkla Health AS	shrimp oil	Norway	EPA 0.4, DHA 0.6	Vitamin E 3 mg, Vitamin D 10 µg, Vitamin A 250 µg	adults and children over 12 years: 5 ml	liquid, 250 ml	EPA and DHA contribute to the normal function of the heart (a beneficial effect is achieved with a daily intake of 250 mg of EPA and DHA). Vitamin E contributes to the protection of cells from oxidative stress. Vitamin D contributes to the maintenance of normal bones. Vitamins A and D contributes to the normal function of the immune system	Dietary supplements are not a substitute for a balanced and varied diet and a healthy lifestyle	The recommended daily dose must not be exceeded. Intended for children over 12 years old and adults
**71**	ORTHOMOL fertil plus tbl	Orthomol	fish oil	Germany	EPA 0.09, DHA 0.06	Vitamin E 40 mg	1	capsules, 30	Omega-3 fatty acids have a beneficial effect on the elasticity of the sperm membrane, which is important for fertilization. Vitamin E contributes to the protection of cells from oxidative stress	Supplement is not a substitute for regular food. This supplement is used as food for special medical purposes. It is used as part of a varied, balanced diet and a healthy lifestyle	Use only under the supervision of a doctor
**72**	ORTHOMOL femin	Orthomol	fish oil, flax seed oil, evening primrose oil	Germany	EPA 0.01, DHA 0.024, ALA 0.025, GLA 0.0068	Vit E 13.5 mg, Vit D 5 µg, Vit C 60mg, Zn 2.5 mg, Se 15 µg, Niacin 10 mg, Pantothenic acid 36 mg, Vit B6 1.5mg, Folic acid 150 µg, Vit B12 3.7 µg, coenzim Q10 5mg	2	capsules, 60, 36 g	Important micronutrients for the health of skin, hair and nails. Vitamin B6 contributes to hormone regulation. Vitamin C and D contribute to the normal maintenance of bones and cartilage.	Nutritional supplements cannot be used as a substitute for a varied and balanced diet and a healthy lifestyle. Keep out of reach of children	The recommended daily dose must not be exceeded

**73**	Pediakid gommes omega 3	Laboratories Ineldea	linseed oil, olive oil	France	ALA 0.0115	Vit E 1 mg, Vit A 60 µg, Vit B5 3 mg, B6 0.7 mg, Zn 0.75 mg	children 2 candies	candies, 60, 138 g	Alpha-linolenic acid favours healthy development and supports cognitive functions. Vit E, B5, B6, A and Zn maintain cognitive functions.	Keep out of reach of children	The recommended daily dose must not be exceeded. It is not recommended for women with a personal or family history of breast cancer. Not suitable for children under 3 years old
**74**	Salvit Omega 3 1000mg	Bee Health Limted	fish oil	United Kingdom	EPA 0.18, DHA 0.12	/	1	capsules, 60, 84.06 g	For heart health	It is important to follow a balanced and varied diet and a healthy lifestyle. Keep out of reach of children	The recommended daily dose must not be exceeded. People who take medication or have special medical conditions should consult a doctor before using this product
**75**	Neuroactive omega and ginkgo and B complex	Lifeline	fish oil	Bosnia and Herzegovina (Belgium)	EPA 0.06, DHA 0.25	dry Ginkgo leaf extract 40mg, Niacin 10 mg, Pantothenic acid 6 mg, Thiamin 1.4 mg, Vit B6 2 mg, Riboflavin 1.6 mg, Folic acid 200 µg, Vit B12 1 µg	1	soft capsules, 30, 28.05 g	DHA contributes to the maintenance of normal brain function (this effect is achieved with a daily intake of 250 mg of DHA). B group vitamins contribute to normal psychological function, functioning of the nervous system and normal mental abilities	/	/
**76**	Omega-3 1000mg	Sensilab	fish oil	Slovenia	EPA 0.5, DHA 0.25	/	1	soft capsules, 30, 36.2 g	The image of *the heart* emphasizes health	Dietary supplements are not a substitute for a balanced and varied diet and a healthy lifestyle. Keep out of reach of children	The recommended daily dose must not be exceeded. In case of hypersensitivity or allergy to any of the ingredients of the product, consult a doctor before use
**77**	Ultra cod liver oil omega-3	Vitabiotics	fish oil	United Kingdom	EPA 0.0645, DHA 0.0595	Vitamin E 0.55 mg, Vitamin D 0.6 µg	2	capsules, 60, 84.06 g	EPA and DHA contribute to the normal function of the heart (a beneficial effect is achieved with a daily intake of 250 mg of EPA and DHA.). DHA contributes to the maintenance of normal	Dietary supplements are not a substitute for a balanced and varied diet and a healthy lifestyle. Keep out of reach of children	The recommended daily dose must not be exceeded. In case of pregnancy, breastfeeding, epilepsy, nutritional allergies or allergies to any of the ingredients, it is necessary to consult a doctor. The product is not suitable for children. If

									brain function and maintenance of normal vision (a beneficial effect will be achieved with 250 mg DHA/day). Vitamin D contributes to the protection of cells from oxidative stress. Vitamin E is a powerful antioxidant that protects the body from free radicals and has a beneficial effect on all conditions accompanied by oxidative stress		you are using anticoagulants (e.g. warfarin), you should consult a doctor before using them
**78**	Osteocar e plus	Vitabiotics	fish oil	USA	EPA 0.032, DHA 0.147	Vitamin D 5 µg, Vitamin C 50 mg	1	capsules, 28	DHA contributes to the maintenance of normal brain function and vision (a beneficial effect is achieved with a daily intake of 250 mg of DHA). Vitamin D contributes to the normal absorption of calcium. Vitamin C contributes to the normal collagen function of bones and cartilage	Dietary supplements are not a substitute for a balanced and varied diet and a healthy lifestyle. Keep out of reach of children	If you are pregnant, breastfeeding, have epilepsy or are under medical supervision, persistent allergies to food or any of the ingredients of the supplement, consult a doctor or pharmacist before use. This product contains Omega-3 fatty acids, and patients using anticoagulants should consult a doctor before use
**79**	Multi Omega 3	Alkaloid	fish oil	Macedonia	EPA 0.188, DHA 0.125	Vit D 2.5 µg, Zinc 1.5 mg, Iodine 22.5 µg, Vit B1 0.27 mg, Vit B2 0.3 mg, Niacin 4 mg, Vit B6 0.3 mg, Biotin 10 µg, Folic acid 37.5 µg, Vit K1 10 µg	5 ml	liquid, 250 ml	DHA contributes to the maintenance of normal brain function and vision. Zinc and iodine contribute to normal cognitive function. Iodine, vitamins B1, B2, B3, B6, B12 and biotin contribute to the normal functioning of the nervous system and brain. Vitamins B2, B3, B12, folic acid and pantothenic acid help to reduce fatigue and exhaustion. Vitamin B2, DHA and zinc contribute to maintaining normal vision	/	/

**80**	PreMama	Alkaloid	fish oil	Macedonia (Poland)	EPA 0.043, DHA 0.2	Vitamin E 3.3 mg	1	capsules, 30, 19.8 g	DHA maternal intake contributes to the normal development of the brain and eye of the foetus and breastfed infants	A nutritional supplement is not a substitute or substitute for a balanced diet. It is important to follow a balanced and varied diet and a healthy lifestyle. Keep out of reach of children	Recommended daily doses must not be exceeded
**81**	Prenatal	Dietpharm	fish oil	Croatia (Greece)	EPA 0.04, DHA 0.2	/	1	capsules, 30, 18 g	DHA maternal intake contributes to the normal development of the brain and eye of the foetus and breastfed infants (a beneficial effect is achieved with an intake of 200 mg of DHA per day in addition to the recommended daily intake for omega-3 fatty acids for adults, i.e. 250 mg of DHA and EPA)	A nutritional supplement is not a substitute or substitute for a balanced diet. It is important to follow a balanced and varied diet and a healthy lifestyle. Keep out of reach of children	Recommended daily doses should not be exceeded. The product is not intended for children under 12 years of age
**82**	Pregnatol DHA	4U Pharma	algae oil	Switzerland	DHA 0.2	/	1	soft capsules, 21 g	DHA maternal intake contributes to the normal development of the brain and eye of the foetus and breastfed infants (a beneficial effect is achieved with an intake of 200 mg of DHA per day)	Nutritional supplements cannot be used as a substitute for a varied diet. Keep out of reach of children	Recommended daily doses must not be exceeded

**83**	Pregnacare plus omega-3	Vitabiotics	fish oil	United Kingdom	EPA 0.06, DHA 0.3	/	1	capsules, 28	DHA maternal intake contributes to the normal development of the brain and eye of the foetus and breastfed infants (a beneficial effect is achieved with an intake of 200 mg of DHA per day)	Nutritional supplements cannot be used as a substitute for a varied diet. Keep out of reach of children	Recommended daily doses must not be exceeded. Do not chew. Use only after meals. If you have a medical condition, epilepsy, thyroid problem, haemophilia, allergy to food or any of the ingredients of the product, consult a doctor or pharmacist before use. This supplement contains Omega-3 fatty acids, and patients using anticoagulants should consult a doctor before use
**84**	DHA mini drops	Natures Aid	fish oil	United Kingdom	EPA 0.1, DHA 0.35	/	children 3 m - 1 year: 0.3 ml (10 drops), 1-5 years: 1 ml	liquid, 50 ml	DHA contributes to the maintenance of normal brain function and vision (a beneficial effect is achieved with a daily intake of 250 mg of DHA). The intake of DHA contributes to the normal development of infants up to 12 months (a beneficial effect is achieved with a minimum intake of 100 mg of DHA). EPA and DHA contribute to the normal function of the heart (a beneficial effect is achieved with a minimum intake of 250 mg of EPA and DHA per day)	Nutritional supplements cannot be used as a substitute for a varied and balanced diet. Keep out of reach of children	Recommended daily doses should not be exceeded. The product should not be taken by people who are hypersensitive to any of the ingredients of the product. Supplements based on fish oil can affect the lowering of blood pressure and the prolongation of coagulation, so they are not recommended for children on anticoagulant therapy and are not applied two weeks before planned surgical procedures. For children who take aspirin, non-steroidal anti-inflammatory drugs, as well as children who suffer from haemophilia and diabetes, they should consult a paediatrician before taking supplements with fish oil
**85**	Femibion 2	P&G Health Austria	fish oil	Austria	DHA 0.2	Vitamin E 11 mg, Lutein from the marigold flower 9 mg	1	capsules, 28	DHA maternal intake contributes to the normal development of the brain and eye of the foetus and breastfed infants (a beneficial effect is achieved with an intake of 200 mg of DHA per day in addition to the	Nutritional supplements cannot be used as a substitute for a varied and balanced diet. Keep out of reach of children.	Recommended daily doses should not be exceeded. The product should not be taken by people who are hypersensitive to any of the product's ingredients. Supplements based on fish oil can affect blood pressure lowering and prolong coagulation, so they are not

									recommended daily intake for omega-3 fatty acids for adults, i.e. 250 mg of DHA and EPA)		recommended for people on anticoagulant therapy and should not be used two weeks before planned surgical procedures. People who take aspirin, nonsteroidal anti-inflammatory drugs, as well as people who suffer from haemophilia and diabetes, should consult a doctor before taking supplements with fish oil. The product is not intended for children under 6 years of age
**86**	Mama plus Doppel herz	Quiesser Pharma	fish oil	Germany	DHA 0.1	Vit E 6.5 mg, Vit D 2.5 µg, Vit C 55 mg, Vit B1/B2/B6/B12 0.6 mg/0.75 mg/0.95 mg/1.75 µg, Biotin 30 µg, Folic acid 300 µg, Niacin 7.5 mg, Ca 100 mg, Mg 45 mg, Zn 5 mg, I 50 µg	1	capsules, 30, 38.4 g	DHA maternal intake contributes to the normal development of the brain and eye of the foetus and breastfed infants (a beneficial effect is achieved with an intake of 200 mg of DHA per day in addition to the recommended daily intake for omega-3 fatty acids for adults, i.e. 250 mg of DHA and EPA)	Nutritional supplements cannot be used as a substitute for a varied and balanced diet and a healthy lifestyle. Keep out of reach of children	Recommended daily doses must not be exceeded
**87**	Mama folic acid and DHA	Mivolis DM	fish oil	Germany	EPA 0.05, DHA 0.25	/	1	capsules, 30	DHA maternal intake contributes to the normal development of the brain and eye of the foetus and breastfed infants (a beneficial effect is achieved with 200 mg DHA/day in addition to the recommended daily intake for omega-3 fatty acids for adults, i.e. 250 mg of DHA and EPA)	Nutritional supplements cannot be used as a substitute for a varied and balanced diet and a healthy lifestyle. Keep out of reach of children	Recommended daily doses must not be exceeded

**88**	Omega-3 Algenol	Mivolis DM	algae oil	Germany	/	/	1	capsules, 30, 21.6 g	EPA and DHA contribute to the normal function of the heart (the beneficial effect is achieved with a daily intake of 250 mg of EPA and DHA.). DHA contributes to the maintenance of normal brain function and maintenance of normal vision (the beneficial effect will be achieved with a daily intake of 250 mg of DHA)	Vegan! An ideal addition to a vegetarian or vegan diet and represents a plant-based alternative to fish-based supplements. Nutritional supplements cannot be used as a substitute for a varied and balanced diet and a healthy lifestyle. Keep out of reach of children	Recommended daily doses must not be exceeded
**89**	Vitup baby D3+DHA	Galenika	algae oil	Serbia (EU)	DHA 0.1	Vitamin D 10 µg	8-day-old infants and small children 1	twist-off capsules, 30, 7.5 g (without capsules)	Vitamin D contributes to the normal growth and development of children's bones and to the normal function of the immune system in children	Nutritional supplements cannot be used as a substitute for a varied and balanced diet. Keep out of reach of children	The recommended daily dose must not be exceeded. The product should not be taken by people who are hypersensitive to any of the ingredients. Pregnant and lactating women should consult a doctor or pharmacist before using this product
**90**	Omega-3 + Vitamin E	Livsane	fish oil	Czech Republic	EPA 0.26, DHA 0.17	Vitamin E 12 mg, Vitamin B6 4.2 mg, Folic acid 600 µg, Vitamin B12 7.5 µg	1	soft capsules, 60, 80.5 g	EPA and DHA contribute to the normal function of the heart (a beneficial effect is achieved with a daily intake of 250 mg of EPA and DHA). Vitamin E contributes to the protection of cells from oxidative stress	Nutritional supplements cannot be used as a substitute for a varied and balanced diet. Keep out of reach of children	The recommended daily dose must not be exceeded. The product should not be taken by people who are hypersensitive to any of the ingredients. supplements based on fish oil can lower blood pressure and prolong coagulation, so they are not recommended for people on anticoagulant therapy and should not be used two weeks before planned surgical procedures. People taking aspirin, nonsteroidal anti-inflammatory drugs, as well as people suffering from haemophilia and diabetes should consult a doctor before taking fish oil supplements

**91**	MOLLERS Ommega 3 Kids	Orkla Health AS	cod liver oil	Norway	EPA 0.4, DHA 0.6	Vitamin E 3 mg, Vitamin D 10mg, Vitamin A 250 µg	children 3-12 years 5 ml	liquid, 250 ml	EPA and DHA contribute to the normal function of the heart (the beneficial effect is ensured by a daily intake of 250 mg of EPA and DHA). DHA provides a positive influence on the normal functioning of the brain and vision. Vitamin D is necessary for the growth and development of bones in children. Vitamin D also contributes to normal immune function	Nutritional supplements cannot be used as a substitute for a varied and balanced diet and a healthy lifestyle. Keep out of reach of children	The recommended daily dose must not be exceeded
**92**	Heart and Mind Easy swallow mini omega 3	Weber Naturals	fish oil	Canada	EPA 0.18, DHA 0.12	/	3	soft capsules, 180	It supports the health of the cardiovascular system and brain function. It helps to reduce serum triglycerides. It helps conventional therapy to reduce rheumatoid arthritis pain in adults	Nutritional supplements cannot be used as a substitute for a varied and balanced diet. Keep out of reach of children	/
**93**	Omega3 1000	Pharmanova	fish oil	Serbia (Germany)	EPA 0.18, DHA 0.12	Vitamin E 10 mg	1	capsules, 30, 41,2 g	EPA and DHA contribute to the normal function of the heart (a beneficial effect is achieved with a daily intake of 250 mg of EPA and DHA). Vitamin E contributes to the protection of cells from oxidative stress	Nutritional supplements cannot be used as a substitute for a varied and balanced diet. Keep out of reach of children	The recommended daily dose must not be exceeded. People sensitive to some of the ingredients should not use the product. Caution is required in people who are on anticoagulant therapy, in people prone to bleeding and in diabetics. People on therapy should consult a doctor
**94**	Omega3 Recharge yourself	Battery	fish oil	United Kingdom (Slovenia)	EPA 0.235, DHA 0.165	/	3	capsules, 90, 149.4 g	Supporting joints and improving general health	/	/
**95**	Sens omega	Sensilab	fish oil	Slovenia	EPA 0.14, DHA 0.095	/	2	capsules, 60, 84.06 g	EPA and DHA contribute to the normal function of the heart (a beneficial effect is achieved with a daily intake of 250 mg of EPA and DHA)	Nutritional supplements cannot be used as a substitute for a varied and balanced diet and a healthy	The recommended daily dose must not be exceeded. People who are sensitive or allergic to some of the ingredients or who use some of the drugs should consult a doctor before use

										lifestyle. Keep out of reach of children	
**96**	Gold Omega 3	Olimp Labs	fish oil	Poland (Poland)	EPA 0.33, DHA 0.22	/	2	capsules, 60	It has a beneficial effect on the cardiovascular system. It affects the reduction of LDL cholesterol and triglycerides and the growth of HDL cholesterol, which reduces the possibility of blood clotting, thus protecting the heart and cardiovascular system	/	/
**97**	Save oil	Inpharm	shrimp oil	Serbia (Norway)	/	Astaxanthin 0.05mg, Phospholipids 200mg	1	capsules, 30, 22.5 g	For maintaining normal functions of the cardiovascular, nervous and locomotor systems as well as preserving vitality	Nutritional supplements are not a substitute for a varied and balanced diet. Keep out of reach of children	Do not exceed the recommended daily doses. There are no known side effects. People allergic to crustaceans must not use this product. In case of hypersensitivity, stop the use of the product immediately

Table S2. Health claims referring to omega-3 fatty acids

Article/ Claim type	Claims on the supplement	Conditions and restrictions for the use of health claims (according to the List of approved health claims)				Number of supplements
		Food requirement	Information for users	Warning for safe intake level	Restriction for use	
	**Claims referring to EPA+DHA**					
Art.13(1)	EPA and DHA contribute to the normal function of the heart (a beneficial effect is achieved with a daily intake of 250 mg of EPA and DHA)	Food which is at least a source of EPA and DHA*	The beneficial effect is obtained with a daily intake of 250 mg of EPA and DHA	-	-	24 supplements for adults, of which one also for children and one for both children and pregnant/nursing women
Art.13(1)	EPA and DHA contribute to the normal function of the heart	Food which is at least a source of EPA and DHA*		-	-	2 supplements, of which 1 for children and 1 for adults
Art.13(1)	DHA and EPA contribute to the maintenance of normal blood triglyceride levels	Food which provides a daily intake of 2 g of EPA and DHA	The beneficial effect is obtained with a daily intake of 2 g of EPA and DHA	Not to exceed a supplemental daily intake of 5 g of EPA and DHA combined	The claim shall not be used for foods targeting children	-
Art.13(1)	DHA and EPA contribute to the maintenance of normal blood pressure	Food which provides a daily intake of 3 g of EPA and DHA	The beneficial effect is obtained with a daily intake of 3 g of EPA and DHA	Not to exceed a supplemental daily intake of 5 g of EPA and DHA combined	The claim shall not be used for foods targeting children	-
	**Claims referring to DHA**					
Art.13(1)	DHA contributes to maintenance of normal brain function	Food which contains at least 40 mg of DHA per 100 g and per 100 kcal	The beneficial effect is obtained with a daily intake of 250 mg of DHA	-	-	3 supplements for adults
Art.13(1)	DHA contributes to the maintenance of normal vision			-	-	-
Art.13(1)	DHA contributes to the maintenance of normal blood triglyceride levels	Food which provides a daily intake of 2 g of DHA and which contains DHA in combination with EPA	The beneficial effect is obtained with a daily intake of 2 g of DHA	Not to exceed a supplemental daily intake of 5 g of EPA and DHA combined	The claim shall not be used for foods targeting children	-
Art.14(1) (b)	Docosahexaenoic acid (DHA) intake contributes to the normal visual development of infants up to 12 months	Food shall contain at least 0,3 % of the total fatty acids as DHA	The beneficial effect is obtained with a daily intake of 100 mg of DHA	-	-	2 supplements for children

Art.14(1)(b)	Docosahexaenoic acid (DHA) maternal intake contributes to the normal development of the eye of the foetus and breastfed infants	Food which provides a daily intake of at least 200 mg DHA	The beneficial effect to pregnant and lactating women is obtained with a daily intake of 200 mg of DHA in addition to the recommended daily intake for omega-3 fatty acids for adults, 250 mg DHA and EPA	-	-	-
Art.14(1)(b)	Docosahexaenoic acid (DHA) maternal intake contributes to the normal brain development of the foetus and breastfed infants			-	-	-
	**Claims referring to ALA**					
Art.13(1)	ALA contributes to the maintenance of normal blood cholesterol levels	Food which is at least a source of ALA	The beneficial effect is obtained with a daily intake of 2 g of ALA and 10 g of LA	-	-	
Art.14(1)(b)	Essential fatty acids are needed for normal growth and development of children	-	The beneficial effect is obtained with a daily intake of 2 g of ALA	-	-	
	**Combined health claims**					
	EPA and DHA contribute to the normal function of the heart (a beneficial effect is achieved with a daily intake of 250 mg of EPA and DHA)	Food which is at least a source of EPA and DHA*	The beneficial effect is obtained with a daily intake of 250 mg of EPA and DHA	-	-	2 supplements for adults
	DHA contributes to the maintenance of normal vision	Food which contains at least 40 mg of DHA per 100 g and per 100 kcal	The beneficial effect is obtained with a daily intake of 250 mg of DHA	-	-	
	EPA and DHA contribute to the normal function of the heart (a beneficial effect is achieved with a daily intake of 250 mg of EPA and DHA)	Food which is at least a source of EPA and DHA*	The beneficial effect is obtained with a daily intake of 250 mg of EPA and DHA	-	-	1 supplement for adults
	Docosahexaenoic acid (DHA) maternal intake contributes to the normal brain development of the foetus and breastfed infants	Food which provides a daily intake of at least 200 mg DHA	The beneficial effect to pregnant and lactating women is obtained with a daily intake of 200 mg of DHA in addition to the recommended daily intake for omega-3 fatty acids for adults, 250 mg DHA and EPA	-	-	
	EPA and DHA contribute to the normal function of the heart (a beneficial effect is achieved with a daily intake of 250 mg of EPA and DHA)	Food which is at least a source of EPA and DHA*	The beneficial effect is obtained with a daily intake of 250 mg of EPA and DHA	-	-	5 supplements for adults

	DHA contributes to maintenance of -normal brain function	Food which contains at least 40 mg of DHA per 100 g and per 100 kcal		-	-	
	-normal vision			-		
	EPA and DHA contribute to the normal function of the heart (a beneficial effect is achieved with a daily intake of 250 mg of EPA and DHA)	Food which is at least a source of EPA and DHA*	The beneficial effect is obtained with a daily intake of 250 mg of EPA and DHA	-	-	1 supplement for adults
	ALA contributes to the maintenance of normal blood cholesterol levels	Food which is at least a source of ALA	The beneficial effect is obtained with a daily intake of 2 g of ALA and 10 g of LA			
	EPA and DHA contribute to - the normal function of the heart	Food which is at least a source of EPA and DHA*	The beneficial effect is obtained with a daily intake of 250 mg of EPA and DHA	-	-	1 supplement for adults
	-the maintenance of normal blood pressure	Food which provides a daily intake of 3 g of EPA and DHA	The beneficial effect is obtained with a daily intake of 3 g of EPA and DHA	Not to exceed a supplemental daily intake of 5 g of EPA and DHA combined	The claim shall not be used for foods targeting children	
	-the maintenance of normal blood triglyceride levels	Food which provides a daily intake of 2 g of EPA and DHA	The beneficial effect is obtained with a daily intake of 2 g of EPA and DHA			
	EPA and DHA contribute to - the normal function of the heart	Food which is at least a source of EPA and DHA*	The beneficial effect is obtained with a daily intake of 250 mg of EPA and DHA	-	-	1 supplement for adults
	-the maintenance of normal blood pressure	Food which provides a daily intake of 3 g of EPA and DHA	The beneficial effect is obtained with a daily intake of 3 g of EPA and DHA	Not to exceed a supplemental daily intake of 5 g of EPA and DHA combined	The claim shall not be used for foods targeting children	
	-the maintenance of normal blood triglyceride levels	Food which provides a daily intake of 2 g of EPA and DHA	The beneficial effect is obtained with a daily intake of 2 g of EPA and DHA			
	DHA contributes to the maintenance of - normal blood triglyceride levels	Food which provides a daily intake of 2 g of DHA and which contains DHA in combination with EPA	The beneficial effect is obtained with a daily intake of 2 g of DHA			
	- normal brain function	Food which contains at least 40 mg of DHA per 100 g and per 100 kcal	The beneficial effect is obtained with a daily intake of 250 mg of DHA	-	-	
	EPA and DHA contribute to the normal function of the heart (a beneficial effect is achieved with a daily intake of 250 mg of EPA and DHA)	Food which is at least a source of EPA and DHA*	The beneficial effect is obtained with a daily intake of 250 mg of EPA and DHA	-	-	1 supplement for adults and children

	DHA contributes to maintenance of normal brain function	Food which contains at least 40 mg of DHA per 100 g and per 100 kcal	The beneficial effect is obtained with a daily intake of 250 mg of DHA	-	-	
	EPA and DHA contribute to the normal function of the heart (a beneficial effect is achieved with a daily intake of 250 mg of EPA and DHA)	Food which is at least a source of EPA and DHA*	The beneficial effect is obtained with a daily intake of 250 mg of EPA and DHA	-	-	1 supplement for children
	DHA contributes to maintenance of -normal brain function	Food which contains at least 40 mg of DHA per 100 g and per 100 kcal	The beneficial effect is obtained with a daily intake of 250 mg of DHA	-	-	
	- normal vision			-	-	
	Docosahexaenoic acid (DHA) intake contributes to the normal visual development of infants up to 12 months of age	Food shall contain at least 0,3 % of the total fatty acids as DHA	The beneficial effect is obtained with a daily intake of 100 mg of DHA	-	-	
	EPA and DHA contribute to the normal function of the heart (a beneficial effect is achieved with a daily intake of 250 mg of EPA and DHA)	Food which is at least a source of EPA and DHA*	The beneficial effect is obtained with a daily intake of 250 mg of EPA and DHA	-	-	2 supplements, 1 of which for children and adults and 1 for children, adults and pregnant/nursing women
	DHA contributes to maintenance of -normal brain function	Food which contains at least 40 mg of DHA per 100 g and per 100 kcal	The beneficial effect is obtained with a daily intake of 250 mg of DHA	-	-	
	- normal vision			-		
	Docosahexaenoic acid (DHA) maternal intake contributes to the normal -development of the eye of the foetus and breastfed infants	Food which provides a daily intake of at least 200 mg DHA	The beneficial effect to pregnant and lactating women is obtained with a daily intake of 200 mg of DHA in addition to the recommended daily intake for omega-3 fatty acids for adults, 250 mg DHA and EPA	-	-	
	-brain development of the foetus and breastfed infants			-	-	
	EPA and DHA contribute to the normal function of the heart (a beneficial effect is achieved with a daily intake of 250 mg of EPA and DHA)	Food which is at least a source of EPA and DHA*	The beneficial effect is obtained with a daily intake of 250 mg of EPA and DHA	-	-	1 supplement for children
	DHA contributes to maintenance of -normal brain function	Food which contains at least 40 mg of DHA per 100 g and per 100 kcal	The beneficial effect is obtained with a daily intake of 250 mg of DHA	-	-	
	- normal vision			-		

	Docosahexaenoic acid (DHA) maternal intake contributes to the normal development of the eye of the foetus and breastfed infants	Food which provides a daily intake of at least 200 mg DHA	The beneficial effect to pregnant and lactating women is obtained with a daily intake of 200 mg of DHA in addition to the recommended daily intake for omega-3 fatty acids for adults, 250 mg DHA and EPA	-	-	
	Docosahexaenoic acid (DHA) intake contributes to the normal visual development of infants up to 12 months of age	Food shall contain at least 0,3 % of the total fatty acids as DHA	The beneficial effect is obtained with a daily intake of 100 mg of DHA			
	DHA contributes to maintenance of -normal brain function	Food which contains at least 40 mg of DHA per 100 g and per 100 kcal	The beneficial effect is obtained with a daily intake of 250 mg of DHA	-	-	2 supplements, of which 1 for children and 1 for adults
	- normal vision			-	-	
	Docosahexaenoic acid (DHA) intake contributes to -the normal visual development of infants up to 12 months	Food shall contain at least 0,3 % of the total fatty acids as DHA	The beneficial effect is obtained with a daily intake of 100 mg of DHA	-	-	1 supplement for children
	-maintenance of normal brain function	Food which contains at least 40 mg of DHA per 100 g and per 100 kcal	The beneficial effect is obtained with a daily intake of 250 mg of DHA	-	-	
	Docosahexaenoic acid (DHA) maternal intake contributes to the normal -brain development of the foetus and breastfed infants	Food which provides a daily intake of at least 200 mg DHA	The beneficial effect to pregnant and lactating women is obtained with a daily intake of 200 mg of DHA in addition to the recommended daily intake for omega-3 fatty acids for adults, 250 mg DHA and EPA	-	-	9 supplements for pregnant/nursing women
	-development of the eye of the foetus and breastfed infants			-	-	

*A claim that a food is a source of omega-3 fatty acids, and any claim likely to have the same meaning for the consumer, can only be made if the product contains at least 0.3 g of ALA per 100 g and per 100 kcal, or at least 40 mg of the total EPA and DHA per 100 g and per 100 kcal.

Table S3. T-test *p*-values for statistical differences* in compliance levels (health claim scores) across the supplements grouped by the country of origin

Country of origin	Austria[b]	Bosnia and Hercegovina	Bulgaria[a]	Canada	Croatia[b]	Chez Republic[b]	EU	France	Germany	Hungary[a]	Italy	Macedonia[b]	Norway	Poland	Romania[b]	Serbia	Slovenia	Spain[a]	Sweden	Switzerland[b]	Taiwan[a]	United Kingdom	USA	Unknown[a]
	p-values																							
Austria[b]	/	0.4405	/	0.6036	/	/	0.4226	0.4226	0.4235	/	**0.0267**	/	0.4918	0.4950	/	0.5587	0.4226	/	**0.0000**	/	/	0.2109	0.6036	/
Bosnia and Hercegovina	0.4405	1.0000	/	0.6147	0.2622	0.2622	0.8652	0.9798	0.9371	/	0.2722	0.3373	0.3493	0.8931	0.4405	0.4336	0.7580	/	**0.0489**	0.3373	/	0.4243	0.4183	/
Bulgaria[a]	/	/	/	/	/	/	/	/	/	/	/	/	/	/	/	/	/	/	/	/	/	/	/	/
Canada	0.6036	0.6147	/	1.0000	0.4468	0.4468	0.8412	0.6813	0.5924	/	0.1088	0.5165	0.8234	0.5948	0.6036	0.8881	0.4891	/	**0.0112**	0.5165	/	0.1866	0.7797	/
Croatia[b]	/	0.2622	/	0.4468	/	/	0.1778	0.1778	0.2584	/	**0.0006**	/	0.3273	0.2856	/	0.4027	0.1778	/	**0.0000**	/	/	0.0780	0.4468	/
Chez Republic[b]	/	0.2622	/	0.4468	/	/	0.1778	0.1778	0.2584	/	**0.0006**	/	0.3273	0.2856	/	0.4027	0.1778	/	**0.0000**	/	/	0.0780	0.4468	/
EU	0.4226	0.8652	/	0.8412	0.1778	0.1778	1.0000	0.8600	0.8888	/	0.1917	0.2722	0.6129	0.8224	0.4226	0.7114	0.7220	/	**0.0220**	0.2722	/	0.4807	0.6036	/
France	0.4226	0.9798	/	0.6813	0.1778	0.1778	0.8600	1.0000	0.9373	/	0.3233	0.2722	0.4059	0.9404	0.4226	0.5179	0.8378	/	0.0599	0.2722	/	0.6079	0.4443	/
Germany	0.4235	0.9371	/	0.5924	0.2584	0.2584	0.8888	0.9373	1.0000	/	0.1967	0.3273	0.3332	0.8212	0.4235	0.3999	0.6765	/	**0.0194**	0.3273	/	0.2396	0.4067	/
Hungary[a]	/	/	/	/	/	/	/	/	/	/	/	/	/	/	/	/	/	/	/	/	/	/	/	/
Italy	**0.0267**	0.2722	/	0.1088	**0.0006**	**0.0006**	0.1917	0.3233	0.1967	/	1.0000	**0.0040**	**0.0072**	0.4586	**0.0267**	**0.0276**	0.5631	/	**0.0748**	**0.0040**	/	0.5937	**0.0217**	/
Macedonia[b]	/	0.3373	/	0.5165	/	/	0.2722	0.2722	0.3273	/	**0.0040**	/	0.3979	0.3739	/	0.4710	0.2722	/	**0.0000**	/	/	0.1264	0.5165	/
Norway	0.4918	0.3493	/	0.8234	0.3273	0.3273	0.6129	0.4059	0.3332	/	**0.0072**	0.3979	1.0000	0.3318	0.4918	0.9369	0.2148	/	**0.0001**	0.3979	/	**0.0409**	0.8722	/
Poland	0.495	0.8931	/	0.5948	0.2856	0.2856	0.8224	0.9404	0.8212	/	0.4586	0.3739	0.3318	1.0000	0.4950	0.4206	0.8915	/	0.1423	0.3739	/	0.6447	0.4138	/
Romania[b]	/	0.4405	/	0.6036	/	/	0.4226	0.4226	0.4235	/	**0.0267**	/	0.4918	0.495	/	0.5587	0.4226	/	**0.0000**	/	/	0.2109	0.6036	/
Serbia	0.5587	0.4336	/	0.8881	0.4027	0.4027	0.7114	0.5179	0.3999	/	**0.0276**	0.4710	0.9369	0.4206	0.5587	1.0000	0.3099	/	**0.0008**	0.4710	/	0.0689	0.8430	/
Slovenia	0.4226	0.758	/	0.4891	0.1778	0.1778	0.722	0.8378	0.6765	/	0.5631	0.2722	0.2148	0.8915	0.4226	0.3099	1.0000	/	0.1830	0.2722	/	0.8318	0.2966	/
Spain[a]	/	/	/	/	/	/	/	/	/	/	/	/	/	/	/	/	/	/	/	/	/	/	/	/
Sweden	**0.0000**	**0.0489**	/	**0.0112**	**0.0000**	**0.0000**	**0.0220**	0.0599	**0.0194**	/	**0.0748**	**0.0000**	**0.0001**	0.1423	**0.0000**	**0.0008**	0.1830	/	1.0000	**0.0000**	/	0.1714	**0.0008**	/
Switzerland[b]	/	0.3373	/	0.5165	/	/	0.2722	0.2722	0.3273	/	**0.0040**	/	0.3979	0.3739	/	0.471	0.2722	/	**0.0000**	/	/	0.1264	0.5165	/
Taiwan[a]	/	/	/	/	/	/	/	/	/	/	/	/	/	/	/	/	/	/	/	/	/	/	/	/
United Kingdom	0.2109	0.4243	/	0.1866	0.078	0.078	0.4807	0.6079	0.2396	/	0.5937	0.1264	**0.0409**	0.6447	0.2109	0.0689	0.8318	/	0.1714	0.1264	/	1.0000	0.1004	/
USA	0.6036	0.4183	/	0.7797	0.4468	0.4468	0.6036	0.4443	0.4067	/	**0.0217**	0.5165	0.8722	0.4138	0.6036	0.843	0.2966	/	**0.0008**	0.5165	/	0.1004	1.0000	/
Unknown[a]	/	/	/	/	/	/	/	/	/	/	/	/	/	/	/	/	/	/	/	/	/	/	/	/

* - ($p \leq 0.05$); [a] - groups of samples with one representative ; [b] - group of samples with no variation in HC scores (all scores = 1)

Table S4. T-test *p*-values for statistical differences* in compliance levels (health claim scores) across the groups classified by the source of omega-3 fatty acid

Omega-3 fatty acid source	algae oil	fish oil	plant oil	fish and plant oil
	p-values			
algae oil	1	0.6087	0.2052	0.2635
fish oil	0.6087	1	**0.0224**	0.3481
plant oil	0.2052	**0.0224**	1	0.0515
fish and plant oil	0.2635	0.3481	0.0515	1

* - ($p \leq 0.05$)

Table S5. T-test *p*-values for statistical differences* in compliance levels (health claim scores) across the groups classified by the target populations

Target population	adult	children	pregnant women
	p-values		
adult	1	0.3034	**0.0291**
children	0.3034	1	0.0810
pregnant women	**0.0291**	0.0810	1

* - ($p \leq 0.05$)

Table S6. Assessment of the omega-3 fatty acid intake expressed as percentage contribution in relation to recommended intake of EPA and DHA

	Recommended intake DHA 100 mg				Recommended intake EPA+DHA 250 mg				
	All	Algae	Fish	Plant	All	Algae	Fish	Plant	Fish+plant
	Infants and 1 y children				2-18 y children				
N	8	3	4	1	21	4	15	2	0
min	0	100	97	0	0	40	120	0	-
max	350	100	350	0	600	40	600	0	-
mean	115.8	100	224.6	0	243.9	40	309.7	0	-
STD	95.7	0	111.0	0	169.9	0	131.5	0	-
median	100	100	222.8	0	244	40	312	0	-
	Infants all				2-3 y toddlers				
N	6	3	2	1	11	3	6	2	0
min	0	100	97	0	0	40	120	0	-
max	350	100	350	0	400	40	400	0	-
mean	97	100	184	0	109.2	40	194.3	0	-
STD	78.5	0	117.4	0	113.8	0	105.0	0	-
median	100	100	105	0	80	40	146	0	-
	Infants 1-3 m				4-10 y children				
N	4	3	0	1	16	1(a)	13	2	0
min	0	100	-	0	0	-	138	0	-
max	100	100	-	0	600	-	600	0	-
mean	75	100	-	0	259.1	-	306.2	0	-
STD	43.3	0	-	0	164.4	-	132.3	0	-
median	100	100	-	0	244	-	250.4	0	-
	Infants 3-6 m				11-14 y younger adolescents				
N	5	3	1	1	16	1(a)	13	2	0
min	0	100	105	0	0	-	138	0	-
max	105	100	105	0	600	-	600	0	-
mean	81	100	105	0	291	-	339.0	0	-
STD	40.5	0	0	0	162.8	-	120.4	0	-
median	100	100	105	0	318	-	342	0	-
	Infants 6-12 m				15-18 y older adolescents				
N	6	3	2	1	15	1(a)	12	2	0
min	0	100	97	0	0	-	138	0	-
max	350	100	350	0	600	-	600	0	-
mean	124.5	100	224	0	282.2	-	333.5	0	-
STD	107.2	0	127	0	166.0	-	124.3	0	-
median	100	100	223.5	0	312	-	324	0	-
	1 y children				Adults				
N	8	3	4	1	73	3	62	5	3
min	0	100	146	0	0	132	23.0	0	27.2
max	350	100	350	0	600	132	600	0	202
mean	157	100	265.2	0	193.0	132	215.7	0	112.8
STD	114.8	0	87.0	0	125.5	0	125.5	0	71.4
median	100	100	300	0	132	132	187.2	0	109.2

(a) – dose not available

Comment S1

Assessment of the contribution of omega-3 fatty acids intake from supplements relative to the intake defined in the consumer information accompanying health claims for omega-3 fatty acids

Since it can reasonably be assumed that the presence of a health claim increases the likelihood of a consumer choosing a supplement over others with the same active substance but without health claims, supplements carrying authorized health claims for ω-3-FA are separated from the supplements without such claims and assessed for their contribution to ω-3-FA intake relative to the intake defined in the consumer information accompanying the health claim for ω-3-FA.

Out of 39 supplements intended for adults that carry a claim regarding EPA/DHA's contribution to normal heart function, 38 provide an intake that matches the amount defined in the consumer information, with an average intake of 232±117%. For one supplement there was not enough labelled data for evaluation. Two supplements with the same claim, but also intended for pregnant and breastfeeding women, fully provide the intake defined in the consumer information (with an average EPA/DHA intake of 200%).

Supplements intended for adults with claims regarding DHA's contribution to normal brain function (13 supplements) and normal vision (10 supplements) provide an average DHA intake of 103±55% (brain) and 109±65% (vision) relative to the amount defined in the consumer information. Individually, six supplements from the brain function group and four from the vision group provide less than 100% (with a minimum of 47.6% in both groups), while one from the brain function group and two from the vision group did not have sufficient labelled data for evaluation. One supplement intended for pregnant women carried both claims and provided 80% of the intake defined in the consumer information.

All five supplements intended for children with the claim regarding DHA contribution to normal visual development in infants up to 12 months provide an intake nearly equal to or greater than the amount defined in the accompanying consumer information (one supplement, designed for children aged 6 months to 18 years, provides 98% of the intake defined for the 6-12 months age group).
